# Applications of Pharmacometrics in Antibody–Drug Conjugate Development

**DOI:** 10.3390/pharmaceutics18030354

**Published:** 2026-03-12

**Authors:** Xiaoliang Cheng, Shuangmin Ji, Yonghyun Lee, Haiyan Dong

**Affiliations:** 1 Department of Pharmacy, The First Affiliated Hospital of Xi’an Jiaotong University, Xi’an 710061, China; chengxiaoliang@mail.xjtu.edu.cn; 2 College of Pharmacy, Ewha Womans University, Seoul 03760, Republic of Korea; 3 GSK, Beijing 100025, China; shuangmin.x.ji@gsk.com; 4 Graduate Program in Innovative Biomaterials Convergence, Ewha Womans University, Seoul 03760, Republic of Korea

**Keywords:** antibody-drug conjugate, pharmacokinetics/pharmacodynamics (PK/PD), modeling, exposure-response (E-R) analysis, dose optimization

## Abstract

Antibody–drug conjugates (ADCs), which integrate a cytotoxic drug known as the payload into a tumor-targeting monoclonal antibody via a linker, have emerged as promising candidates for cancer therapy and are a new avenue for targeted cancer therapy. The pharmacokinetic (PK) profiles of ADCs are distinctive due to their unique distribution, catabolism, and elimination. Their deconjugation in circulation and variations in the drug-to-antibody ratio increase the complexity of their PK profiles. Pharmacometric models depicting the PK properties and exposure-response (E-R) relationships of ADCs are important for optimizing dosing regimens and supporting decisions during ADC development. This review considers the PK profiles of ADCs, physiologically based PK models, semi-mechanistic and mechanistic PK models, population PK models, and E-R analyses for dose optimization. The prospects and challenges for ADCs, especially the urgent need for advanced analytical technology and modeling approaches, are also outlined.

## 1. Introduction

Antibody–drug conjugates (ADCs), which tether a cytotoxic small-molecule agent known as the payload to a tumor-targeting monoclonal antibody via a linker, have emerged as promising candidates for cancer therapy and are a new path for targeted cancer therapy [1]. The concept of using ADCs as “biological missiles” dates back to the early 20th century, when Paul Ehrlich proposed that toxins be attached to antibodies to enhance therapeutic specificity [2]. The advent of hybridoma technology in 1975, and the subsequent emergence and development of monoclonal antibodies (mAbs) that specifically target cancer cytomembrane antigens, have promoted the realization of “biological missiles” [3]. ADCs, which are composed of a tumor-targeting mAb linked to a cytotoxic payload via a linker, achieve precise targeting, potent efficacy, and limited uptake in antigen-negative cells, and their therapeutic applicability is widening [4]. The first ADC, Mylotarg^®^ (gemtuzumab ozogamicin), was approved by the U.S. FDA in 2000 for adults with acute myeloid leukemia. Since then, 13 ADCs have been approved by the U.S. FDA and about 100 ADCs are going through clinical trials, which has greatly encouraged academic research and the development of novel ADCs [5].

**Figure 1:**
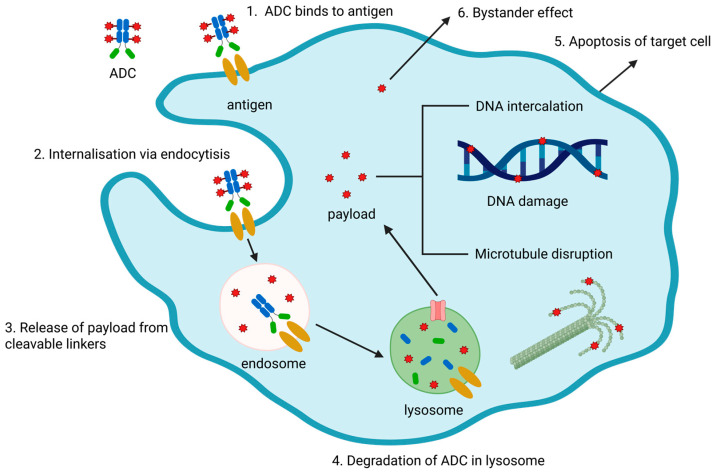
Mechanistic actions of ADCs. Created in BioRender. Cheng, X. 2026. BioRender.com/xell8ja.

ADCs are a heterogeneous modality due to dynamic changes in the average number of small-molecule drug particles attached to each mAb, which is designated as the-drug-to-antibody ratio (DAR). After administration, the mAb facilitates specific targeting to antigen-expressing carcinoma cells, leading to internalization of the ADC via receptor-mediated endocytosis. ADCs combine the specific targeting behavior of an mAb with the tumoricidal efficacy of a cytotoxic agent [6]. Upon internalization, the payload is released from the ADC via hydrolytic or proteolytic cleavage of the linker, which is responsive to low pH or the reductive environment of cytoplasm and enzymes, and the unconjugated cytotoxic drug reaches its action site (e.g., microtubules, DNA) and displays its pharmacological potency [7]. This accounts for the target-mediated drug disposition (TMDD) characteristic of ADCs and alters the PK of the cytotoxic drug. The released cytotoxic drug diffuses into the cancer cells and triggers cell apoptosis or death, primarily by damaging DNA construction or microtubule polymerization [8]. This targeted drug delivery achieves a high concentration of the cytotoxic payload in tumor cells while minimizing systemic exposure and off-target toxicity. However, if the liberated payload is permeable or transmembrane, some of it can diffuse into the extracellular matrix and access neighboring cells, whether or not they express the targeting antigen, which is designated as the bystander effect [9]. The bystander effect enhances the cytotoxic potency of an ADC to cells that are deficient in the targeting antigen, particularly in highly heterogeneous cancer lesions with different expression levels of the targeted antigen [10]. In addition, the antibody component can boost tumor cell death via other pathways, such as antibody-dependent cellular cytotoxicity, antibody-dependent cellular phagocytosis, and complement-dependent cytotoxicity [11]. The mechanistic action of ADCs is illustrated in Figure 1. Those unique pharmacological mechanisms endow ADC monotherapy with exceptional clinical efficacy, showing 30–90% objective response rates with manageable safety issues [12,13,14].

Despite promising progress in ADC development, these products face great challenges. The primary challenge is the need to concurrently optimize and characterize all three entities that compose an ADC (mAb, cytotoxic drug, and linker). Correspondingly, investigating and anticipating the pharmacokinetics (PK) and pharmacodynamics (PD) of ADCs is challenging because they demand simultaneous depiction of PK and PD profiles for an mAb, a small-molecule agent, and their conjugate. In addition, antibodies are not catalyzed by cytochrome P450 enzymes or eliminated by the kidney or liver because their molecular weight is too large to diffuse through podocyte fenestration of the kidney glomeruli or be a substrate for hepatic efflux transporters [15,16]. Decomposition of mAb primarily depends on the endocytic system, with vasculature in the liver and spleen serving as the dominant sites of degradation [15,17]. After they are internalized into cancer cells by receptors, ADCs are subjected to degradation into small peptides and amino acids through hydrolysis or lysosomal enzymes [18]. Deconjugation, which describes the liberation of the cytotoxic agent via cleavage of the linker, occurs in both target cells and systemic circulation [19]. Deconjugation and DAR variation collectively contribute to the complexity of PK profiles for ADCs, highlighting the need and challenge of in vivo quantification of multiple analytes (ADCs with different DARs, total antibody, free antibody, and released drug). However, tumor concentration is inaccessible, which undermines the potency of models to depict PK behaviors in tumor. Both catabolism and deconjugation can occur simultaneously. Saturable target-regulated elimination gives the ADC nonlinear PK behavior. The ADC prolongs the half-life of the payload by delaying its release, which enhances its efficacy [20]. The payload released from the ADC is mainly subjected to metabolism in the liver and then excreted from the body via urine or feces, eliciting the risk of drug–drug interactions [21].

Modeling the PK of ADCs is complicated because they have unique and various elimination routes (metabolism and deconjugation), and ADCs with different DARs, unconjugated antibodies, and the liberated drug all possess distinct PK profiles. Pharmacometrics is a critical multidisciplinary discipline gathering PK and PD data, and mathematical models, further providing a quantitative framework for understanding, characterizing, and predicting in vivo behavior, drug exposure and response, drug–drug interaction, and optimization of therapeutic strategies [22,23,24]. A wide array of pharmacometrics models encompassing physiologically based, semi-mechanistic and mechanism-based and population PK models for ADCs have been proposed. Furthermore, optimizing the dosing regimen during clinical development and therapy is pivotal for therapeutic efficacy, tolerability, and reducing toxicities such as peripheral neuropathy and cytopenia. An extensive understanding of their PK properties and exposure–response (E-R) relationships is explored to optimize dosing regimens and support clinical decisions about ADCs.

This review considers physiologically based PK (PBPK) models, semi-mechanistic and mechanistic PK models, population PK models, and E-R analyses for dose justification. Relevant studies were retrieved from PubMed through title/abstract searches using combinations of “PK model” which includes the aforementioned models and “antibody–drug conjugate”. It also analyzes the developments and challenges in ADC PK-PD investigations, explores a prospective orientation for academic investigation, and proposes tactics to solve remaining problems. Moreover, we provide proposals and ideas for PK-PD research and individualized therapy using ADCs.

## 2. Pharmacokinetics Models for Antibody–Drug Conjugates

### 2.1. Physiologically Based Pharmacokinetics Models for Antibody–Drug Conjugates

#### 2.1.1. Introduction for Physiologically Based Pharmacokinetics Models

Traditional mechanism-based PK profiles cannot adequately describe ADCs. Given their complicated molecular constitution and unique disposition, catabolism, and elimination profiles, it is imperative to incorporate all the processes that determine the disposition of ADCs and their constituents into a PK model, particularly the intracellular procedures responsible for drug release and exposure inside the cells. PBPK models offer a precious opportunity to understand the whole-body disposition of ADCs and the relationship between their exposure and efficacy or exposure and toxicity.

Generally, the drug concentration in plasma or serum, which is easily accessible, can be used to represent the drug concentration at the active site. However, for ADCs, the plasma or serum concentration alone is insufficient to develop the E-R and exposure–toxicity relationships, so a comprehensive understanding of the PK in organs, especially the tumor, is urgently needed [25]. However, quantifying ADCs in organs has great challenges, and current analytical methodologies cannot feasibly measure the whole-body distribution of ADCs in humans. Therefore, it is indispensable to establish a model that is competent to anticipate whole-body PK, especially PK in tumor to establish reliable E-R relationships, and PBPK models can meet those requirements. PBPK models are mathematical models composed of multiple compartments that describe and predict PK profiles based on a drug’s physical and chemical characteristics and mechanistic processes that incorporate measurable physiological parameters at the organism level [26]. PBPK model arrangements are more complex than traditional models because the multiple compartments represent the total physiology of the organism and describe the absorption, distribution, metabolism, and excretion (ADME) of the drug and its metabolites. Whole-body PBPK models consist of explicit compartments that represent all the organs and tissues involved in the ADME of the drug and its metabolites. Other parameters for each organ, such as blood flow, volume, tissue-partition coefficient, and permeability, are also featured [27]. A perfusion- or permeability-rate limited model is leveraged to describe drug transportation. A perfusion-rate limited model assumes that the tissue membrane is not a barrier for drug delivery, so the velocity of blood flow is the rate-limiting aspect. In a permeability-rate limited model, drug-specific permeability, rather than the velocity of blood flow, is the rate-limiting aspect [28]. Arterial and venous blood compartments are used to connect organs and tissues. PBPK models are useful for extrapolating PK parameters to different species, populations, or disease statuses. They are closely associated with each species’ physiology and the drug physicochemical properties, and they can also incorporate inter-individual variabilities such as sex, age, ethnicity, genetic polymorphisms, and varied physiological characteristics in different disease statuses or subpopulations to evaluate individual PK variability. Therefore, PBPK models are appropriate for ADCs with complicated PK profiles and provide precious insights for establishing E-R relationships for ADCs. Because they are a powerful strategy for predicting the concentrations of a drug and its metabolites in organs, regulatory agencies have accepted PBPK models for investigations instead of dedicated clinical trials [29]. 

#### 2.1.2. Application of Physiologically Based Pharmacokinetics Models in Antibody–Drug Conjugates Development

The primary application for PBPK models is predicting DDI potential in lieu of a dedicated DDI clinical trial [30]. DDIs involving the antibody constituent of an ADC are limited in use because that component is catabolized via nonspecific proteolytic decomposition and TMDD, with no prominent involvement of CYPs. However, the released cytotoxic drug could be metabolized and excreted by CYPs and transporters. Therefore, the investigation of DDIs for ADCs mainly focuses on the cytotoxic agent. A PBPK model could incorporate and explain the mechanism for the DDI of interest. Unlike an empirical compartment model, a mechanism-based PBPK model achieves a “what-if” scenario analysis, especially if there is a knowledge gap about the latent molecular mechanisms and experimental data are scarce. For example, predicting the PK of released monomethyl auristatin E (MMAE), which is a microtubule-disrupting agent, via linker cleavage and assessing its associated DDIs is a great challenge because the mechanisms and kinetics of the cleavage have not been fully elucidated [31,32]. Those gaps can be bridged by integrating “bottom-up” and “top-down” approaches in a mechanistic PBPK structure that uses parameters from in vitro studies and computerized predictions (“bottom-up”) and clinical data (“top-down”). So in this example, in silico, in vitro, and in vivo PK data from an ADC consisting of an anti-CD22 antibody, MMAE, and valine-citrulline (vc) linker were combined in a mixed “bottom-up” and “top-down” methodology to establish a PBPK model linking the antibody-conjugated MMAE to its catabolite, unconjugated form. Because this vc-MMAE ADC possessed the same linker as another ADC, it was possible to leverage knowledge from a previous DDI study of an ADC containing vc-MMAE to inform the appraisal of this vc-MMAE ADC. Model validation was performed using clinical PK data for brentuximab vedotin (another vc-MMAE ADC), and the validated model was applied to simulate the consequence of DDIs between brentuximab vedotin and midazolam, ketoconazole, and rifampicin. The ratios of the area under the concentration–time curve (AUC) and maximum concentration (C_max_) simulated by the proposed model were within two-folds of the observed clinical DDI data [33]. The resulting PBPK model could be extended to assess the DDI potential of other vc-MMAE ADCs.

**Figure 2:**
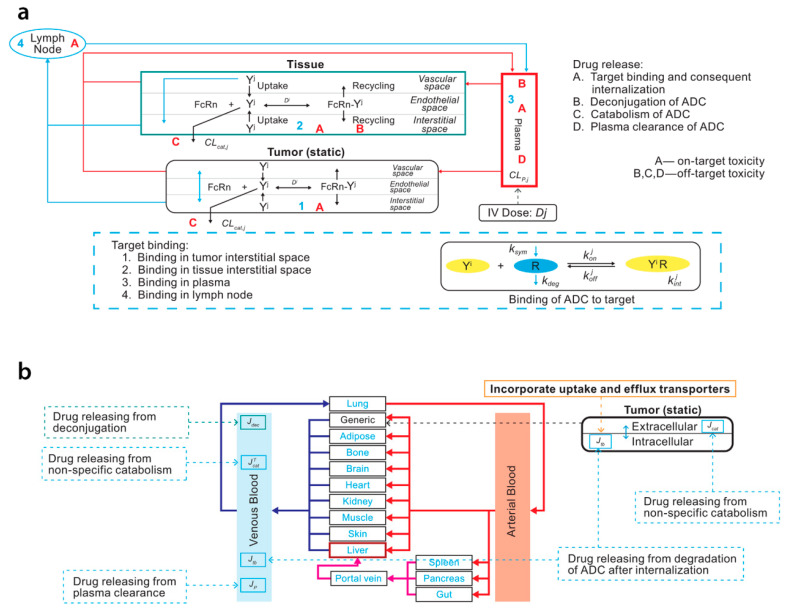
Schematic illustration of (**a**) the Simcyp simulator ADC module (**b**) incorporated into a full PBPK model for the payload [34]. (Reprinted with permission from Certara Limited). Note: IV, intravenous; *Dj*, dose for ADC with DAR of j; FcRn, neonatal Fc receptor; DAR, drug-to-antibody ratio; j, DAR number; Y^j^, ADC with DAR of j; ${CL}_{p,j}$, plasma clearance for ADC with DAR of j; *CL_cat,j_*, catabolic clearance for ADC with DAR of j; *D^j^*, association and dissociation of ADC and FcRn; R, antigen; $k_{sym}$ and $k_{deg}$, synthesis and degradation rate constant of antigen; $k_{on}^{j}$ and $k_{off}^{j}$, association and dissociation rate constant of ADC–antigen complex, respectively.

A PBPK model was developed to anticipate the influence of ketoconazole, as an inhibitor of both P-glycoprotein and cytochrome P450 3A4 (CYP3A4), and rifampin, as a combined P-glycoprotein and CYP3A4 inducer, on the PK of enfortumab vedotin, which consists of a Nectin-4-directed antibody and MMAE and is mainly eliminated via P-glycoprotein-regulated excretion and CYP3A4-catalyzed metabolism. In addition, the influence of enfortumab vedotin on exposure to midazolam and digoxin, which are substrates for CYP3A4 and P-glycoprotein, was analyzed separately. The Simcyp simulator, which uses a minimal PBPK modeling approach, was applied to construct an ADC module for the PBPK model, and a full PBPK approach was leveraged for the cytotoxic agent. The neonatal Fc receptor dissociation constant was optimized for enfortumab vedotin based on clinical data. The results of this PBPK simulation suggested that a P-glycoprotein and CYP3A4 inhibitor could increase MMAE exposure and that potential adverse effects should be monitored carefully, whereas a P-glycoprotein and CYP3A4 inducer could decrease MMAE exposure. Enfortumab vedotin had no obvious influence on exposure to CYP3A4 or P-glycoprotein substrates [34]. A schematic diagram of the PBPK model is illustrated in Figure 2. Owing to limited data, assumptions were made when creating the conjugated mAb portion of the enfortumab vedotin model. Moreover, MMAE concentration time profiles displayed large variability which could not be fully captured by the model.

Many ADCs share identical payloads and mechanistic PBPK models can extrapolate previous knowledge to new compounds. Based on data from a previous clinical DDI study for brentuximab vedotin as an analog of vc-MMAE, a PBPK was fabricated and validated to assess the CYP3A-regulated DDI potential of polatuzumab vedotin, which is an anti-CD79b-v-MMAE ADC. Similarly to brentuximab vedotin, polatuzumab vedotin was predicted to have DDI potential at the proposed labeled dose in the presence of a potent CYP3A inducer or inhibitor, and it was not expected to act as either an inhibitor or inducer of CYP3A [35]. In the absence of a dedicated DDI clinical trial, the PBPK-based model informed drug development as a powerful method for predicting the CYP3A-regulated DDI for the drug label.

Systemic exposure to an ADC and its liberated drug cannot accurately represent the exposure of individual organs [36,37]. Quantitative and comprehensive investigations of the whole-body PK of ADCs is a prerequisite for establishing E-R relationships and assessing their safety profiles [36]. The whole-body distribution of trastuzumab-vc-MMAE in tumor-bearing mice was quantified by analyzing the total mAb and total and released MMAE using enzyme-linked immunosorbent assays and liquid chromatography–mass spectrophotometry (LC-MS). A PBPK model for the ADC was established by integrating PBPK models for the mAb and small-molecule agent and assuming that deconjugation of the drug occurred in a DAR-dependent manner. The results showed that the ADC deconjugation rate was upregulated in certain tissues, such as the spleen, and that conjugation of the small-molecule drug had no prominent influence on the PK of trastuzumab, but the tissue distribution of MMAE was altered by its conjugation to the mAb. MMAE exposure in the tumor increased by approximately 20 times when it was administered as an ADC, compared with free administration. The proposed PBPK model was able to precisely predict the PK profiles of all three ADC analytes in plasma, tissues, and the tumor and could be scaled up to humans [38].

Trastuzumab emtansine (T-DM1) is composed of a recombinant and humanized anti-human epidermal growth factor receptor 2 (HER-2) monoclonal IgG antibody, a derivative of the microtubule inhibitor maytansine, and a stable nonreducible thioether bond linker, and it is applied to treat HER-2 positive cancers [39]. Trastuzumab is an inhibitor of the PI3K/AKT signaling pathway and HER-2 shedding, and it also can elicit antibody-dependent cellular cytotoxicity [40]. A translational PBPK model for T-DM1 leveraged a previously published rat PBPK model for the antibody and successfully translated DM1 biodistribution data in rats to humans. The resulting human model predicted the plasma PK of T-DM1 and total trastuzumab reasonably well, though a slight prediction bias was observed for DM1. Those results, a pathway analysis, and a global sensitivity analysis collectively suggested that parameters associated with mAb decomposition, ADC deconjugation, and the released agent PK are pivotal for depicting and understanding the whole-body biodistribution of the ADC [41].

A tissue-level PK model that collectively leverages geometry and mixed boundaries can identify whether the drug transportation dynamic is perfusion- or permeability-rate limited, making it a powerful model for describing the tissue-scale distribution of a wide array of drugs [42,43,44]. Integrating a Krogh cylinder into a PBPK model can accurately depict and predict drug distribution in heterogeneous tumor compartments, and those results can also be translated into other species. Cilliers et al. integrated a PBPK model for the whole-body disposition of T-DM1 with a tumor tissue Krogh cylinder to develop a multiscale model that simulated both its systemic distribution and its distribution in a heterogeneous tumor. Their results demonstrated that T-DM1 at a clinical dose (3.6 mg/kg) exhibited highly heterogeneous tumor distribution, representative of perivascular distribution. Coadministration of trastuzumab promoted homogeneous tumor distribution and significantly improved T-DM1 tumor penetration. The dose, deconjugation, DAR, receptor expression, and trafficking all prominently influenced ADC distribution [45].

MMAE, a microtubule-disrupting agent, is one of the payloads most commonly used when fabricating ADCs, and some ADCs containing MMAE, including Adcetris^®^ (brentuximab vedotin), Padcev^®^ (enfortumab vedotin-ejfv), and Polivy^®^ (polatuzumab vedotin-piiq), are approved and marketed [46]. MMAE can induce hematological adverse drug reactions such as neutropenia and thrombocytopenia, and peripheral neuropathy is another major adverse drug reaction for MMAE in clinical trials [47,48]. A PBPK model was developed to depict the whole-body PK of MMAE in tumor-bearing mice. It elucidated perfusion/permeability-limited drug transportation in organs, blood cell distribution, plasma protein binding, and tissue/tumor retention. The proposed model commendably described the PK profiles of MMAE in plasma, organs, and tumors, and the estimated model parameters showed good confidence [47]. However, the PK characters of free MMAE depicted in this study and predicted by the proposed PBPK model may not represent the actual exposure of MMAE after MMAE-conjugated ADC administration, which is due to the mAb delivering cytotoxic drugs and thus altering the PK profiles and targeting potency of the payload. PBPK models for ADCs are summarized in Table 1.

PBPK models require massive amounts of input data about the PK profiles of a drug, and the input parameters obtained from in vitro studies or computerized forecast methodologies are highly variable, so underlying uncertainty and failure of extrapolations remain. Therefore, an ever-increasing accumulation of knowledge about ADCs and persistent optimization of the PBPK model is necessary. Moreover, precise parameters for human physiology have not yet been defined, so in-depth studies are needed to develop more accurate PBPK models.

### 2.2. Semi-Mechanistic and Mechanistic Models for Antibody–Drug Conjugates

#### 2.2.1. Introduction to Semi-Mechanistic and Mechanistic Models

Empirical PK-PD models which utilize classic compartment models have been used to quantitatively describe ADC disposition and efficacy [49,50]. These classic compartment models, which use linear clearance from a central compartment, can be extrapolated to some extent, for instance, across species. However, empirical models cannot describe the relatively complex disposition and pharmacologic action of ADCs. A mechanism-based PK-PD model is urgently needed to characterize the PK profiles and in vivo behaviors of ADCs and their payloads.

A mechanism-based PK-PD model with an applied quantitative approach can be leveraged to understand the mechanistic processes of drug disposition and action [51]. The intricate processes and nonlinear kinetics of the circulation and cellular disposition of ADCs and their components are ideal for mechanistic models [52]. A mechanistic PK-PD model not only comprehends the underlying mechanisms of drug disposition and action but can also be applied to ADC design, optimal ADC selection, preclinical to clinical translation, and the optimization of a dose regimen. Mechanism-based PK models can integrate an understanding of the PK behavior of an ADC and its multiple catabolites, as well as the underlying mechanisms that govern that behavior. Furthermore, these models can predict the PK characteristics of ADCs and their components, which are infeasible to measure in organs, thereby providing powerful information for quantitatively appraising efficacy and toxicity.

#### 2.2.2. Semi-Mechanistic and Mechanistic-Based Models for Antibody–Drug Conjugates

A mechanism-based model was developed to identify the rate and pathway for deconjugation. Affinity capture capillary LC-MS was proposed to quantify individual ADC moieties with different DARs [53]. Bender et al. developed a mechanistic PK model for T-DM1 using PK data from affinity capture LC-MS collected during preclinical in vitro and in vivo studies in rats and cynomolgus monkeys. The mechanistic model conceptualized the degradation of T-DM1 to its individual moieties with different DARs and further quantified their distribution and elimination, along with the DM1 deconjugation rate. ADCs with higher DARs had a faster DM1 deconjugation rate, and the clearance rate for T-DM1 was two times higher than that for total trastuzumab because of deconjugation [39]. An integrated mechanistic model of ADC PK described the explicit behavior of all DAR species, sequential deconjugation dependent on DAR, and DAR-dependent antibody and ADC clearance for MMAE- and monomethyl auristatin F (MMAF)-conjugated ADCs. The PK characteristics of the total antibody, drug-conjugated antibody, and antibody-conjugated drug for different DARs and targets in rodents and cynomolgus monkeys were combined to establish the model. The combined cross-species model successfully captured the PK features of multiple analytes after an administration of pure ADCs with the appointed DARs and ADCs with hybrid DARs. The integrated mechanistic model can be used for a wide array of ADC entities to explore the mechanisms underlying the disposition of the ADC and make cross-species predictions [54]. A mechanism-based TMDD model of load-independent properties was formulated to characterize the PK of ADCs via two coupled two-compartment systems with parallel linear and Michaelis–Menten elimination. Rapid binding (quasi-equilibrium), quasi-steady-state, and Michaelis–Menten approximations for the model were derived. The next step considered the special case of the ADC elimination rate and deconjugation rate for each payload, independent of DAR, and then the model was further simplified under various assumptions. The fully formulated model described clinical PK data well for T-DM1 simulated from published models [55]. Total antibody, antibody-conjugated MMAE, and released MMAE were measured in cynomolgus monkeys that received single and multiple doses of an anti-CD79b-MMAE ADC, and a hybrid, iterative-2-stage, Monte-Carlo expectation–maximization method was applied to develop a semi-mechanistic PK model. The measured total antibody and conjugated and released MMAE were adequately depicted by the final model, which revealed that MMAE was liberated through both proteolytic decomposition and deconjugation, whereas proteolytic degradation appeared to be responsible for the MMAE released into systemic circulation [56].

A multiscale mechanism-based PK-PD model was developed to characterize the disposition of an ADC and its payload at the cellular level and translate preclinical efficacy data to the clinic. Brentuximab vedotin, which consists of an anti-CD30 antibody, the potent tubulin polymerization inhibitor MMAE, and a vc linker, is approved for treating Hodgkin’s lymphoma and anaplastic large cell lymphoma [57]. Brentuximab vedotin is stable in systemic circulation, and MMAE is released via cleavage of the linker by lysosomal cathepsin-B in the target cancer cells [52]. Taking brentuximab vedotin as an example, a multiscale mechanism-based PK-PD model was built for the preclinical to clinical translation of ADC therapeutic efficacy. Brentuximab vedotin experimental data harvested from various publications were leveraged to build and verify a model that characterized ADC and payload PK at the cellular level, payload PK in plasma and xenograft tumors, and ADC PK in plasma and predicted payload concentrations in xenograft tumors. The PK-PD model incorporated a tumor disposition model for the ADC that characterized preclinical tumor growth inhibition data, described the ADC and payload PK in patients with cancer, and predicted the clinical response to brentuximab vedotin. The tumor disposition model precisely anticipated payload concentrations in both plasma and xenograft tumors. The clinical progression-free survival (PFS) rates and complete response rates for brentuximab vedotin predicted by the PK-PD model were comparable to the observed outcomes [52]. A mechanistic model was also developed for an anti-5T4 ADC comprising a humanized anti-5T4 antibody, MMAF, and a protease-resistant maleimidocaproyl linker to a priori predict concentrations of the ADC and its liberated payload in two different xenograft tumors and explore the most sensitive pathways and parameters. The predicted total antibody, ADC, liberated payload, and exposure to the payload in plasma were within two times the observed values. Payload dissociation from the ADC and tumor volume were revealed to be important determinants of payload exposure in plasma and tumors [58].

Accurate characterization of the intracellular disposition of an ADC is of great significance for a priori predictions of cellular unconjugated drug concentrations. Therefore, Singh et al. fabricated a mathematical model to characterize the detailed intracellular disposition of T-DM1 in different HER-2 expressing cell lines, predict DM1 concentrations in a xenograft tumor model that integrated a cellular model with a tumor disposition model, and identify the dominant pathways and sensitive parameters responsible for intracellular activation of the ADC. Their cellular disposition model was further optimized by introducing the intracellular proteolytic decomposition of ADC and passive transport of the released drug across cancer cells. Various analytes quantified in previously published articles were included in the optimized model to describe the in vitro PK of T-DM1 in three HER-2 positive cell lines. The tumor disposition model, which incorporated the cellular model, accurately predicted DM1 concentrations in the xenograft tumors of mice. A mechanistic analysis revealed that antigen-mediated and passive diffusion pathways contributed to intracellular payload exposure both in vitro and in vivo. Global and local sensitivity analyses demonstrated that non-specific deconjugation and passive transportation across neoplastic cytomembranes were the pivotal parameters for intracellular drug exposure [59]. Effective ADC entry and payload release in cancer cells depends on the expression of target antigens on the cytomembrane, so mechanistic models can also be leveraged to explore the influence of antigen expression on ADC disposition. Trastuzumab deruxtecan, consisting of an anti-HER-2 monoclonal IgG1 antibody, a novel camptothecin derivative (deruxtecan) as a topoisomerase I inhibitor, and an enzyme-responsive tetrapeptide-based linker with a mean DAR of 7–8, is approved to treat unresectable or metastatic HER-2 positive breast cancer [2,60]. A step-by-step and mechanism-based preclinical model was developed to capture the totality of E-R data for trastuzumab deruxtecan in mice bearing different xenograft tumors with varying HER-2 expression. The deruxtecan dynamics across cell lines with varying HER-2 levels were determined for trastuzumab deruxtecan, and isotypes with DARs of four and eight and free deruxtecan in vitro were incorporated into the mechanistic model. Making use of plasma PK data, allometric scaling from mice to humans, and variability in tumor growth kinetics, including tumor doubling time and the killing rate induced by the payload, researchers created clinical trial simulations for virtual patients with varying HER-2 expression and identified stratification criteria and applications for various clinical scenarios [61]. Using trastuzumab-vc-MMAE and four breast cancer cell lines with varying HER-2 levels, a mechanism-based PK model was applied to mathematically characterize the PK of total trastuzumab, liberated MMAE, and total MMAE in a tumor xenograft model. The results uncovered an apparent positive correlation between antigen expression and free or total MMAE exposure in tumors but not with total trastuzumab exposure. Understanding the intricate physiology of cancer is urgently needed to predict the PK of ADCs and their components, and clinical expression screening prior to targeted therapies is highly recommended to maximize the therapeutic benefits of ADCs [62].

A mechanistic PK-PD model was proposed to translate preclinical information to the clinic for inotuzumab ozogamicin, a CD22-targeting ADC approved to treat B cell malignancies, including non-Hodgkin’s lymphoma (NHL) and acute lymphocytic leukemia (ALL). The PK-PD model integrated a plasma PK model featuring the disposition and clearance of inotuzumab ozogamicin and its liberated payload (*N*-acetyl-calicheamicin 1, 2-dimethyl hydrazine dichloride), a tumor disposition model describing ADC diffusion into the tumor extracellular environment, and a cellular model characterizing ADC binding to its receptor (CD22), its internalization, intracellular liberation of its payload, binding to DNA, and efflux from the cancer cell, as well as tumor proliferation and suppression in a mouse xenograft model. By introducing human PK profiles of inotuzumab ozogamicin (i.e., clinical tumor volumes, proliferation rates, and CD22 expression levels in patients), the preclinical model was translated to the clinic to predict PFS rates in patients receiving inotuzumab ozogamicin therapy, and the predicted results were comparable to the observed outcomes. The model also indicated that a fractionated dosing regimen would work better than a conventional one for ALL but not for NHL. Simulations revealed tumor growth as a hypersensitive parameter when predicting clinical outcomes, and the ADC PK and payload efflux were more powerful predictors of a clinical response than CD22 receptor expression [63]. The PK of T-DM1 in humans were a priori predicted by using allometric scaling of PK parameters in monkeys. A PK model composed of two integrated two-compartment models was used for simultaneously characterizing the disposition of T-DM1 and DM1 catabolites in the systemic circulation. A multi-scale PKPD model was leveraged to characterize the tumor distribution of T-DM1 and DM1 catabolites and ADC-induced tumor growth killing. With an assumption of a spherical tumor, size/radius of the tumor determined the rate of ADC/drug exchange in the tumor via diffusion across the tumor surface or permeability across the blood vessel endothelium. When the tumor was small, the growth was characterized mainly using the exponentially function *Kg^Ex^*, and as the tumor grew, the model switched to linear growth (*Kg^Lin^*). At a very late stage, the tumor volume reached a maximum carrying capacity (*V_max_*) and the tumor stopped growing. Total intracellular concentrations of unconjugated DM1 catabolites (free and tubulin bound) were used to induce the regress in the tumor volumes (*TV*(*t*)) using a 1st-order killing rate constant (*K*_Kill_). The anticipated PK profiles in humans, preclinically anticipated parameters for therapeutic efficacy, and clinically witnessed dimensions and proliferation parameters for breast carcinoma collectively contributed to the development of a translated clinical PK-PD model for T-DM1. Furthermore, PFS and objective response rates after T-DM1 treatment were predicted by the translated PK-PD model via simulation. The predicted PFS rates for HER-2 1+ and 3+ sub-populations were comparable to the observed ones, and the model uncovered only a modest increment in the objective response rate with an increase in the approved dose of T-DM1. A fractionated dosing regimen was also recommended by the model to improve treatment efficacy [64]. The quality of the predictions by this model heavily relied on the quality of parameter values used to perform clinical simulations. With the goal of enhancing the translation of an ADC’s preclinical therapeutic effects to humans, a novel semi-mechanistic model based on the cell cycle that incorporated the tumor growing fraction was proposed to describe tumor growth. PK and PD data for an anti-glypican 3 ADC consisting of an IgG1 anti-glypican 3 mAb, the anti-tubulin agent tubulysin, and a vc linker in a hepatocellular carcinoma patient-derived xenograft model were used to evaluate a proposed semi-mechanistic model. Tumor static concentrations were ascertained using tumor growth inhibition and PK data. The estimated tumor static concentrations and human PK data translated from cynomolgus monkeys were collectively leveraged to predict an effective clinical dosing regimen of 0.20–0.63 (median 0.5) mg/kg every 3 weeks [65]. PK-PD models incorporating the tumor growing fraction rather than the whole tumor as a proliferative mono-compartment could improve human dose prediction for ADCs and other chemotherapeutic agents.

The influence of the payload, especially its ability to diffuse outside the targeted carcinoma cells into adjacent cells (bystander effect) has not been fully elucidated. A predictive computational model was established to investigate the payload distribution in tumors as a function of antibody dose, payload dose, and payload properties. The proposed model revealed that the tumoral distribution of the payload and the potency of both non-bystander and bystander payloads were improved by enhancing the tumor penetrating capability and direct targeting of carcinoma cells by an ADC. In addition, the enhanced penetration capability and bystander effect could make up for weak penetration of the antibody, and a high dose further improved its penetration capability. The bystander effect is responsible for the death of antigen-negative cells, so the physicochemical properties of the payload should be optimized to achieve quick diffusion into cancer cells, exemption from efflux, and slow arrival at distant cells. In other words, optimization of the antibody dose, payload dose, and physicochemical properties of the payload is essential for targeted delivery and maximum efficacy [66].

**Figure 3:**
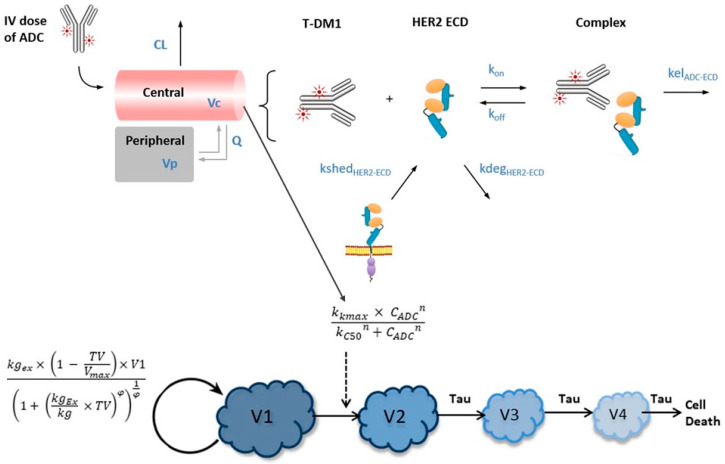
TMDD model used to describe clinical PK of T-DM1 and to predict clinical PK of PF-06804103. Note: IV, intravenous; V_c_, central compartment volume; CL, clearance; V_p_, peripheral compart-ment volume; Q, inter-compartmental clearance; ECD, extracellular domain; k_on_, binding rate con-stant of T-DM1 and HER2 EDC; k_off_, dissociating rate constant of T-DM1 and HER2 EDC; HER2 binding affinity; kshed_HER2-ECD_, rate constant for HER2 shedding; kdeg_HER2-ECD_, rate constant for HER2 degradation; kel_ADC-ECD complex_, elimination rate constant of the HER2-ADC complex; HER2 ECD, concentration of serum HER2 ECD; k_g,Ex_, exponential growth rate; k_g_, linear growth rate; V_max_, maximum growth rate; Tau, transduction time; k_k,max_, maximum kill rate; k_c50_, Concentration at half maximal kill rate; TV, total tumor volume; V1-V4, tumor volume in the growth compartment and three transduction compartments; n, hill co-efficient; Ψ, constant for switching from exponen-tial to linear growth patterns.

Mechanism-based models have also been used to predict and compare the PK profiles of ADCs. As illustrated in Figure 3, a mechanistic, target-mediated drug disposition model using a tumor growth inhibition model was constructed based on T-DM1, which has nonlinear PK, to anticipate the PK profiles of a novel HER-2 ADC PF-06804103 in humans. PF-06804103 was predicted to possess nonlinear PK characteristics, and its PK depended on the circulating HER-2 extracellular domain concentration. A mouse PK-PD model was translated to the clinic and showed that PF-06804103 was more potent than T-DM1 [67]. A combined cross-species model for MMAE- and MMAF-conjugated ADCs was successfully applied to predict the PK profile of anti-six-transmembrane epithelial antigen of the prostate 1-vc-MMAE, and the anticipated data agreed well with the observed total antibody and antibody-conjugated drug concentrations in a dose-ranging phase I clinical trial [54]. A PK-PD model was fabricated to investigate the mechanistic processes of PK, tumor uptake, and catabolism, along with the efficacy of T-DM1 and T-SPP-DM1 (with a distinct cleavable disulfide linker). ^3^H-labeled DM1 facilitated a quantitative analysis of the ADC in plasma and the tumor and catabolites in the tumor. The analytes were fitted using three mechanistic PK-PD models, and the tumor response was linked to the tumor catabolite concentration. The results showed that the DM1 concentration in plasma after T-DM1 administration was eliminated more slowly than after T-SPP-DM1 administration due to a discrepancy in DM1 release. The mechanistic model demonstrated that T-DM1 exhibited a faster tumor catabolism and efflux rate for the catabolite than T-SPP-DM1, which was inconsistent with the expected profiles based on the physicochemistry of their catabolites. Catabolite concentration in the tumor was adequate to describe the antitumor efficacy, and T-DM1 and T-SPP-DM1 possessed similar potency [68]. Semi-mechanistic and mechanistic-based PK models for ADCs are demonstrated in Table 2.

Mechanistic models are based on the known physiological processes governing the disposition of ADCs and their payload within the tumor; however, the in vivo behavior of ADCs is not fully elucidated and many processes and parameters are assumed. So developing a reliable mechanism-based model is dependent on the development of basic research on tumor biology and cellular disposition of ADCs. It is hypothesized that mechanistic models can be applied to predict tumor concentrations of ADCs and catabolites in patients, which is instrumental in the selection of optimum dose and precision medicine. However, the predicted results of allometric scaling models need to be rigorously verified.

### 2.3. Population Pharmacokinetics Models for Antibody–Drug Conjugates

Since the approval of brentuximab vedotin in 2011, population PK models have generally been used to assess the clinical PK profiles of ADCs for dose optimization [69]. Given heterogeneous DAR and various catabolites for ADCs, the choice of analytes used to develop a population PK model should be considered carefully to ensure that the proposed model can accurately describe the PK of the ADC. One-, two-, and three-analyte-based population PK models have been reported. This section introduces population PK models for ADCs, with an emphasis on mechanism-based population PK models.

#### 2.3.1. Single-Analyte-Based Population Pharmacokinetics Models for Antibody–Drug Conjugates

Due to the assumption that the concentration of the conjugated antibody, rather than the payload and other catabolites, is closely related to the efficacy and toxicity of an ADC, the conjugated antibody is used to construct population PK models. Two-compartment models with first-order [70,71] or parallel linear and Michaelis–Menten elimination kinetics [72] were developed for conjugated T-DM1, and covariate screening recommended a weight-based dosing regimen. A two-compartment model with integrated linear and time-dependent clearance, which represented the target-mediated drug disposition pathway fitted with a concentration–time profile in adult and pediatric patients, was developed for inotuzumab ozogamicin. The proposed model indicated that no adjustment of the initial dosing regimen was needed [73,74]. A single-analyte model possesses the advantages of an oversimplified structure, ease of application, and minimum data requirement. However, it is unable to fully capture the complex PK of ADCs.

#### 2.3.2. Two-Analyte-Based Population Pharmacokinetics Models for Antibody–Drug Conjugates

Two-analyte models for ADCs combine the conjugated antibody and released cytotoxic agent or integrate the conjugated antibody and total antibody. Incorporating the conjugated antibody and total antibody undermines the descriptive and predictive capability of two-analyte models due to their close correlation. The merits of joining the conjugated antibody and free payload lie in the mechanistic elucidation of elimination pathways for the ADC and the yield of free agent in vivo.

**Figure 4:**
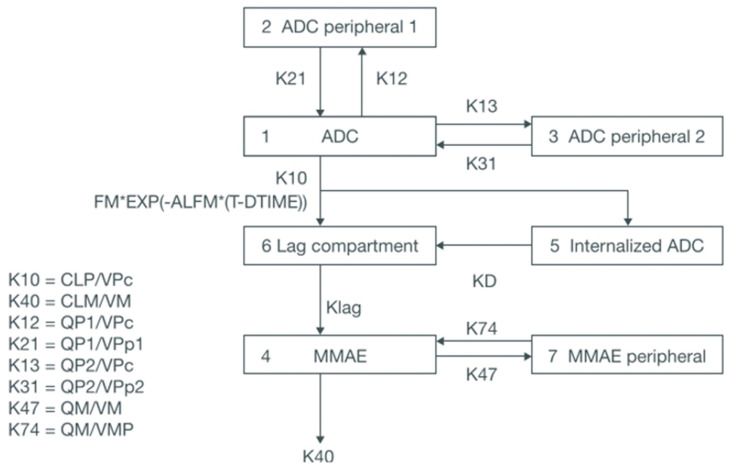
Population PK model with a lag compartment for brentuximab vedotin and MMAE. (Reproduced with permission from Ajit Suri et al. (2018). Copyright 2018 John Wiley and Sons [75]). Note: ADC, antibody–drug conjugate; FM, fraction metabolized; ALFM, rate constant to describe the decline in direct conversion of ADC to MMAE following time after dose; *, multiplication; CLM, apparent MMAE clearance; CLP, ADC clearance; KD, binding rate constant; K_lag_, rate constant for lag compartment; QM, apparent MMAE intercompartmental clearance; QP1 and QP2, ADC intercompartmental clearance from the central to the first and second peripheral compartments, respectively; VM and VMP, apparent volume of the MMAE central and peripheral compartments, respectively; VPc, volume of the ADC central compartment; VPp1 and VPp2, volume of the ADC first and second peripheral compartments, respectively.

A two-analyte model for brentuximab vedotin in patients with cutaneous T-cell lymphoma leveraged a three-compartment model: the PK of released MMAE was described using a two-compartment model with first-order elimination, and a lag compartment was used to depict the delayed generation of MMAE from its direct release and liberation after binding to its target. A covariate analysis uncovered that the body surface area had a prominent effect on the clearance of MMAE and the ADC, and that the central distribution volume and clearance of the ADC decreased as the albumin concentration increased [75]. The same population model for brentuximab vedotin was applied for dose optimization in patients with Hodgkin’s lymphoma to manage peripheral neuropathy and neutropenia [76] and in both pediatric and adult patients to compare body surface area-based dosing scenarios in pediatric patients with a body weight-based dosing regimen in adults [77]. The PK model encompassing a lag compartment for brentuximab vedotin and MMAE is demonstrated in Figure 4. A similar model for brentuximab vedotin that considered the simultaneous production of free MMAE via degradation and deconjugation of the conjugated antibody was constructed by Li et al. and revealed that body weight was a significant factor for PK [78]. A linear three-compartment model fit the brentuximab vedotin PK well, and an empirical sigmoidal function was used to eliminate the ADC and interpret the time-varying generation rate of MMAE. The model indicated that the PK profiles of brentuximab vedotin, administered as monotherapy or as a portion of an integrated regimen, in pediatric patients were consistent with those in adults and offered a foundation for a body weight-based dosing regimen of 1.8 mg/kg every 3 weeks irrespective of age [79]. An integrated linear three-compartment model for brentuximab vedotin with zero-order input and first-order elimination and two compartments for released MMAE was constructed to identify the factors affecting the PK of brentuximab vedotin and MMAE. A lag compartment was included to characterize the delayed yield of MMAE. The resulting population PK model and E-R analysis recommended a dose of 48 mg/m^2^ every 2 weeks for brentuximab vedotin in combination with adriamycin, vinblastine, and dacarbazine for pediatric patients with newly diagnosed advanced Hodgkin’s lymphoma [80].

Yin et al. described trastuzumab deruxtecan PK by integrating a two-compartment model with linear elimination for intact trastuzumab deruxtecan and a one-compartment model with a time-dependent release-rate constant and linear elimination for the liberated cytotoxic agent. Their results offered no dose optimization recommendations for specific populations [81].

An integrated population PK model for T-DM1 was developed based on T-DM1 conjugate and total trastuzumab serum concentration data that encompassed the baseline trastuzumab concentration before T-DM1 therapy in phase I and II clinical trials. Based on the assumption that T-DM1 degraded to trastuzumab via one-step deconjugation, a two-compartment model for T-DM1 and trastuzumab was added. The final model showed that T-DM1 elimination routes included both deconjugation and proteolytic decomposition. The model precisely anticipated the total trastuzumab exposure for a further E-R analysis of T-DM1 PK profiles and used a sparse sampling tactic for total trastuzumab [82]. Beginning with a quantification of the conjugated and total antibody, a semi-mechanistic population PK model described the deconjugation processes of T-DM1 from high to low DAR using five two-compartment models with different DARs to predict the clearance of each conjugated antibody from the central compartment. A simulation by the proposed model demonstrated that the time-concentration profile for T-DM1 with a higher DAR was lower than that for T-DM1 with a lower DAR due to an enhanced clearance rate for T-DM1 with a higher DAR. The semi-mechanistic model accounted for the underlying mechanism of an extended half-life for the total antibody, compared with the conjugated one, caused by the deconjugation process [83]. A two-compartment PK model with parallel linear and Michaelis–Menten elimination pathways was constructed for the total and conjugated antibody of camidanlumab tesirine, which is composed of a human IgG1 anti-CD25 mAb, pyrrolobenzodiazepine dimer cytotoxin SG3199, and a cleavable linker. The baseline level of soluble CD25 was negatively associated with exposure, female patients exhibited higher exposure than male ones, and patients with high body weights had higher exposures than those with low body weights. The PK model revealed that deconjugation of the conjugated antibody accounted for a prominent proportion of the ADC clearance, and clearance was amplified by a time-dependent mechanism [84].

A population PK model that concurrently depicted the concentrations of antibody-conjugated and free MMAE was constructed after multiple administrations of polatuzumab vedotin in non-Hodgkin’s lymphoma patients. A two-compartment model comprising nonspecific time-dependent linear clearance, linear time-dependent exponentially declining clearance, and nonlinear Michaelis–Menten clearance fit the antibody-conjugated MMAE concentration in plasma well. Nonspecific time-dependent linear clearance was representative of systemic/extracellular clearance, intracellular clearance, endocytosis, pinocytosis, and neonatal Fc receptor-mediated clearance, and the other two routes reflected target-mediated drug disposition. All of the elimination routes were responsible for generating free MMAE in the central compartment. Liberated MMAE was described by a two-compartment model with apparent clearance and nonlinear Michaelis–Menten clearance. The complete model verified the current weight-based individualization of the dosing regimen [85]. It was also successfully applied to population PK and an E-R analysis of polatuzumab vedotin in patients with previously untreated diffuse large B-cell lymphoma [86].

Tisotumab vedotin, consisting of an anti-tissue factor antibody and MMAE, was rapidly approved by the U.S. FDA in 2021 to treat recurrent or metastatic cervical cancer [87,88]. A two-compartment population PK model for tisotumab vedotin with parallel linear and Michaelis–Menten elimination and a delay compartment and a one-compartment model for free MMAE generation and elimination were constructed separately in phase I/II trials. The input to the delay compartment was calculated by multiplying the clearance rate for the conjugated antibody by DAR over time. Similarly to the brentuximab vedotin model, released MMAE was found to result from nonspecific elimination of the ADC and the delay compartment. Body weight was the most significant covariate affecting the distribution and elimination of the ADC and MMAE, offering evidence for a weight-based dosing regimen [89].

Based on phase I data, a semi-mechanistic two-analyte population PK model was constructed for a novel, targeted HER-2 ADC, FS-1502. Two-compartment PK models with parallel linear and nonlinear Michaelis–Menten elimination and first-order elimination were leveraged to describe the PK of FS-1502 and unconjugated MMAF, respectively. The model supported a body weight-based dosing regimen for FS-1502. The population PK model and E-R analysis were a quantitative and holistic model-informed tactic to ascertain the recommended phase II dose for FS-1502 instead of using the traditional maximum tolerated dose approach [90].

#### 2.3.3. Three-Analyte-Based Population Pharmacokinetics Models for Antibody–Drug Conjugates

A three-analyte model simultaneously depicts the PK profiles of the conjugated antibody, total antibody, and liberated cytotoxic agent and explores in-depth mechanisms for the in vivo behavior of the ADC. However, this model is usually complex, and extensive clinical data, a meticulous sampling scheme, and specific bioanalytical methods are required.

In 2020, the U.S. FDA and the European Medicines Agency approved belantamab mafodotin, composed of a monoclonal antibody targeting B-cell maturation antigen on malignant multiple myeloma plasma cells and the cytotoxic drug MMAF, to treat multiple myeloma [88,91]. Rathi et al. constructed a semi-mechanistic population PK model consisting of a linear two-compartment model with first-order and time-dependent clearance depicted by a sigmoidal time function for the conjugated and total antibody and a two-compartment model with first-order elimination for the payload released in patients with multiple myeloma. MMAF released into the central compartment was produced by degrading and deconjugating clearance pathways for the conjugated antibody, and the yield of liberated MMAF was manipulated by time-dependent DAR. The model identified baseline soluble B-cell maturation antigen, sex, albumin, and body weight as prominent covariates for exposure to the conjugated antibody and released MMAF [92]. Other models characterized belantamab mafodotin PK by leveraging a linear, two-compartment model with decreasing clearance over time that was described by a sigmoidal time function and characterized the PK of liberated MMAF using a linear two-compartment model in which the MMAF input rate was mediated by proteolytic ADC degradation, which was regulated by exponentially declining DAR after each dose [93,94].

Loncastuximab tesirine is an ADC consisting of an anti-CD19 antibody and the chemotherapeutic drug SG3199, which attacks DNA cross-linking in target cells; it is used to treat relapsed or refractory diffuse large B-cell lymphoma [95]. In 2021, loncastuximab tesirine became the first CD19-targeting ADC to receive rapid approval in the United States. A population PK model for loncastuximab tesirine was composed of a two-compartment linear model with time-dependent clearance of the conjugated and total antibody and a one-compartment model with linear elimination of the liberated SG3199. The model comprehensively described the release of SG3199 via a deconjugation process and its elimination via the liver and kidney and also screened out significant covariates for exposure [96].

**Figure 5:**
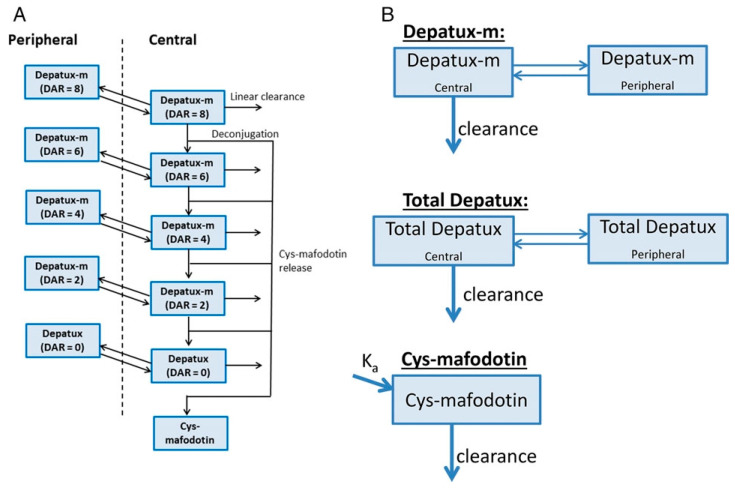
Schematic representation of a structural PK model for depatuxizumab mafodotin. (**A**) The integrated ADC model, (**B**) individual models. (Reproduced with permission from Rajendar K Mittapalli et al. (2019). Copyright 2019 John Wiley and Sons [97]). Note: DAR, drug-to-antibody ratio; K_a_, first-order absorption rate; Depatux-m, depatuxizumab mafodotin; Depatux, depatuxizumab; cys-mafodotin, cysteine-mafodotin.

A two-compartment model for each DAR of depatuxizumab mafodotin, an anti-epithelial growth factor (EGFR) ADC, with linear clearance for unconjugated antibody, and a one-compartment model for its payload, cys-mafodotin, collectively described the concentration–time data for the ADC and its catabolites. Linear PK profiles verified the hypothesis that depatuxizumab mafodotin did not prominently bind to wild EGFR in normal organs. The visual predictive checks demonstrated that the model accurately fit the ADC, total antibody, and cys-mafodotin PK [97]. The structure of the PK model for depatuxizumab mafodotin is illustrated in Figure 5.

A sequential modeling strategy was used to construct a population PK model for enfortumab vedotin. A linear three-compartment model with first-order elimination for enfortumab vedotin was preliminarily modeled, and a two-compartment model with first-order elimination for released MMAE and the exponentially time-dependent conversion of MMAE from the ADC was input into the model. Simulations showed that a weight-based dosing regimen generated more identical exposures than a hypothetical fixed-dose regimen [98].

Hibma et al. developed a population PK model for gemtuzumab ozogamicin that consisted of a two-compartment model with linear and time dependent clearance, including a decay coefficient for the antibody, and a two-compartment model with an input rate of production dependent on the elimination rate of the ADC. It revealed that mildly or moderately impaired renal or hepatic function did not alter the antibody disposition [99]. This PK model was applied to pediatric patients, and body weight was identified as a significant covariate affecting the PK of gemtuzumab ozogamicin in pediatric patients, but when body size was taken into consideration, no recommendation for dose adjustment for age was needed [100].

Mirvetuximab soravtansine is a first-in-class ADC-targeting folate receptor α that received approval for treating platinum-resistant ovarian cancer [101]. Tu et al. constructed a semi-mechanistic population PK model for the ADC, its payload, and a metabolite of the payload. The structural configuration was a two-compartment model with zero-order input (intravenous infusion) and first-order and Michaelis–Menten elimination: a target compartment representing the tumor for the production and disposition of the payload (DM4) and its metabolite (S-methyl DM4) and a central compartment for SG3199 and its metabolite. The model revealed that no adjustment of the recommended dose of 6 mg/kg was required in patients with mildly or moderately impaired renal function or mildly impaired hepatic function, and weak and moderate CYP3A4 inhibitor levels had no significant influence on exposure to mirvetuximab soravtansine [102].

Patritumab deruxtecan is composed of a fully human mAb against HER-3, a topoisomerase I inhibitor payload, and a tetrapeptide-based cleavable linker [103]. The PK of patritumab deruxtecan were analyzed using a two-compartment model with parallel linear and nonlinear clearance. The liberation of the payload (deruxtecan, which is a derivative of exatecan) was a first-order and time-dependent function of the concentration of patritumab deruxtecan in the central compartment, and released deruxtecan was characterized by a one-compartment model with linear clearance. The model demonstrated that the influence of covariates on exposure metrics was usually minor, and no dose optimization for subpopulations was needed [104]. A PK model for patritumab deruxtecan comprising a two-compartment model for the ADC (with linear transient clearance representing high clearance in the first cycle, nonspecific time-dependent clearance, and nonlinear clearance) and a one-compartment model with linear and nonlinear clearance to describe the PK of the payload was constructed. The yield of deruxtecan involved all three clearance pathways for ADCs. Exposure to deruxtecan, rather than patritumab deruxtecan, was significantly enhanced by moderate hepatic impairment [105].

Sacituzumab govitecan is an ADC consisting of a Trop-2-directed antibody coupled to SN-38 as a topoisomerase inhibitor via a hydrolyzable linker, and it is approved for breast cancer therapy [106]. The PK of the total antibody was best characterized by a two-compartment model with first-order and time-dependent elimination. The PK of the unconjugated antibody was described using a two-compartment model with first-order linear elimination, and the PK of free SN-38 featured a sequential model for its generation through first-order release from the ADC. The results showed that a body weight-based dosing regimen was applicable to the target population, and further dose adjustment was unnecessary [107]. Population PK models for ADC are presented in Table 3.

The reported population PK models for ADCs also made certain assumptions to accommodate the complexity of their disposition, and some studies included sparse concentration data and small numbers of patients, which may lead to a greater magnitude of variability in some PK parameters. The absence of a description of the target binding of the payload to exert cytotoxicity is also a limitation to the models. The proposed population PK models possess limited generalizability of the investigated ADC to other ADCs targeting identical antigen.

In this section, PBPK models, semi-mechanistic and mechanistic models, and population PK models for ADCs were introduced and discussed. PBPK models can depict the whole-body disposition of ADCs, and the primary application for PBPK models is predicting DDI potential and organic concentrations over time. However, underlying uncertainty and failure of extrapolations remain. Semi-mechanistic and mechanistic models can describe the mechanistic processes of drug disposition and action, and can be leveraged to predict tumor concentrations of ADCs and catabolites in patients. However, due to the fact that many in vivo processes and parameters for ADCs are assumed, the anticipated results of allometric scaling models should be rigorously verified. The population PK model is generally leveraged to assess the clinical PK profiles of ADCs for dose optimization. However, the population PK model is incompetent in depicting the complexity of ADCs disposition.

The core considerations for model selection include several key factors: (1) research stage: PBPK and semi-mechanistic or mechanistic models are preferred in the early development stage to depict whole-body and mechanistic processes of drug disposition and action, while the population PK model is widely leveraged in clinical trials for dose regimen optimization; (2) research purpose: PBPK and semi-mechanistic or mechanistic models are feasible for mechanism interpretation, while the population PK model is suitable for dose optimization and clinical variability interpretation; (3) data availability: PBPK requires massive amounts of input data about the PK profiles of ADCs and their payloads and physiological parameters, and semi-mechanistic or mechanistic models also need abundant data for characterizing the PK profiles and in vivo behaviors. PBPK models and semi-mechanistic and mechanistic models can be combined with population PK to predict tumor concentration profiles for ADCs and their payload in patients for dose regimen optimization. However, this extrapolation should be stringently assessed.

## 3. Exposure-Response Analyses for Antibody–Drug Conjugates

The next step in modeling for ADC development is E-R analyses, which use mathematical functions to characterize the relationship between drug exposure and patient response. The exposure variable is the drug concentration, such as C_max_ and AUC, and the response is usually a clinical endpoint indicating the drug’s safety or efficacy status [108]. E-R analyses, which are highly recommended by the U.S. FDA, are widely leveraged in current drug discovery and development processes to furnish information that can support determinations of safety and efficacy [109]. In March 2024, the U.S. FDA issued a specific guidance for ADC drug development that recommended E-R analyses for ADCs and their components to support dosage selection and optimization [110]. The China Center for Drug Evaluation (CDE) and National Medicine Products Agency (NMPA) issued similar guidance in September 2025, emphasizing the importance of E-R analyses for ADC drug development [111]. Exposure metrics and modeling approaches widely used for ADC E-R analyses are discussed in this section.

### 3.1. Exposure Metric Considerations

The first decision point and challenge for ADC E-R analyses is the choice of appropriate exposure metrics for the ADC and other components of interest. Compared with the parent drug, its active metabolites and mAbs, the complex components of ADCs have more complicated exposure features [112,113]. Although researchers have many choices when selecting the active moieties for an E-R analysis, the U.S. FDA generally suggests that all active moieties should be considered [109]. In ADCs, each element has the potential to affect the overall efficacy and safety. For example, ADC drug concentrations in circulation could represent the stability of the ADC because the antibody elicits the specific binding of the ADC to its target antigen, and the cytotoxic payload exerts cytotoxicity and drives tolerability [2]. Thus, in theory, all the elements should be explored in an E-R analysis. However, in practice, not all the constituent parts of an ADC are explored during the lifecycle of ADC development.

Although the U.S. FDA generally requests E-R analyses for ADCs and their components, in late-phase drug development, sponsors can provide justification for not conducting an E-R analysis for a constituent part of ADC. For instance, the sponsor could waive payload exploration if the payload is still undetectable with a highly sensitive bioanalytical method, or it could waive total antibody exploration if the mAb carrier serves only to selectively deliver the payload and the total antibody concentrations are highly correlated with the ADC [112]. As listed in Table 4, in the E-R analyses performed for all 13 ADCs approved by the U.S. FDA, the exposure metrics of the ADC, total antibody, and payload have been explored for different products. The summarized result demonstrates that for most of the products, ADC and/or payload exposure metrics were selected in E-R models, with exposure of the total antibody used for only a few products, e.g., gemtuzumab ozogamicin [114] and inotuzumab ozogamicin [115].

Different specimens, e.g., serum/plasma, tumor, cerebrospinal fluid, and urine, can be applied to the analysis; systemic exposures in serum/plasma were leveraged in all of the approved ADCs because the exposure data in tumor tissues are inaccessible [116]. As presented in Table 4, the exposure metrics used in E-R analyses for the currently approved ADCs are primarily derived using the empirical Bayes estimate method based on the population PK model, while observed exposure metrics are also applied in the E-R analysis. Observed and model-predicted C_min_ and AUC for trastuzumab emtansine at cycle 1 were tested to explore E-R relationships; however, model-predicted cycle 1 C_min_ showed the strongest E-R trend [117].

Given that various metrics representing exposure levels during different treatment cycles could provide exposure information from different perspectives, the advantages and limitations of different exposure metrics should be fully considered. The exposure metrics commonly used for the approved ADCs dominantly encompass early exposure metrics, steady state exposure metrics, and time-average exposure metrics [118].

Early exposure metrics, e.g., trough concentration in the first cycle (C_trough,C1_), average concentration in the first cycle (C_avg,C1_), and AUC in cycle 1, can be acquired based on the first cycle concentrations. Early exposure metrics can minimize the effects of frequent dose modification in follow-up treatment cycles, which is very common during cancer treatment [119]. Cycle 1 exposure metrics were the most widely used metrics in E-R analyses for approved ADC products such as gemtuzumab ozogamicin, ado-trastuzumab emtansine, and inotuzumab ozogamicin [118]. However, because early exposure metrics provide limited information about changes in clearanceover time or dose modification during treatment, they are inapplicable for longitudinal E-R models, especially when the relative rank of a patient’s exposure changes after long-term treatment [113,120].

Steady state exposures, such as the average steady state concentration (C_avg,ss_) and steady state trough concentration (C_trough,ss_), which match the endpoint after long-term treatment, are landmark metrics for E-R analyses. They were successfully applied to an exposure–safety analysis of brentuximab vedotin and trastuzumab deruxtecan. However, because ADCs display time-dependent and response altered PK properties, linking drug exposure at the steady state to clinical outcomes in a single-dose trial can yield an over-steep E–R relationship [113,120]. Steady state exposures have been shown to lead to potential bias in E-R analyses for several mAb products. A positive AUC_ss_ and efficacy relationship was observed for tremelimumab; however, in a subsequent study an increased dose regimen did not lead to improved efficacy [121].

Time-averaged exposure metrics, which are usually acquired until an event time, are informative because they account for dose interruptions and reductions. For example, the average concentration up to an event time (C_avg,TE_) was successfully applied in an exposure–efficacy analysis for trastuzumab deruxtecan [122]. However, in oncologic trials subjects commonly encounter a censored event. Right-censored events do not occur until the end of study, and left-censored events happen before observations begin with an unknown actual occurrence time [123]. For those subjects, time-averaged exposure metrics can introduce bias, especially when the dropout rate induced by concurrent exposure-related adverse events is high. Wiens et al. simulated a time-to-event response scenario that was independent of exposure and analyzed the simulated dataset using C_avg,TE_ or C_avg,C1_ independently [124]. The results showed that using C_avg,TE_ as a predictor resulted in a clear positive relationship with the response, whereas using C_avg,C1_ led to an unbiased conclusion of no E-R relationship. Similar results were observed by Liu et al. [125]; their simulation for nivolumab showed that using exposure variables observed or derived from the first treatment cycle for an E-R analysis might minimize that bias. As described by Lin et al., using different approaches to derive C_avg,TE_, for example, different time imputation approaches or time-varying derivation methods for censored subjects, can potentially bias the results [126]. Wiens et al. suggested that C_avg,TE_ should be considered as a chief exposure metric for E-R bias, and recommended the use of exposure metrics that are definitely independent of the outcome or intercurrent events, such as cycle 1 exposures, for E-R analyses [124].

### 3.2. Exposure-Response Modeling

As shown in Table 4, currently, the most used approaches for ADC E-R analyses are survival analyses (encompassing Kaplan–Meier plots and Cox proportional hazards models) and logistic regression models.

#### 3.2.1. Survival Analyses

Survival analysis is applied to analyze the duration from treatment initiation to a well-defined event of interest, including safety and efficacy endpoints [127]. The common endpoints for ADC products in oncologic treatment include overall survival, adverse events of interest, disease recurrence, and death. At present, non-parametric, semi-parametric, and parametric methods are used for survival analyses.

**Figure 6:**
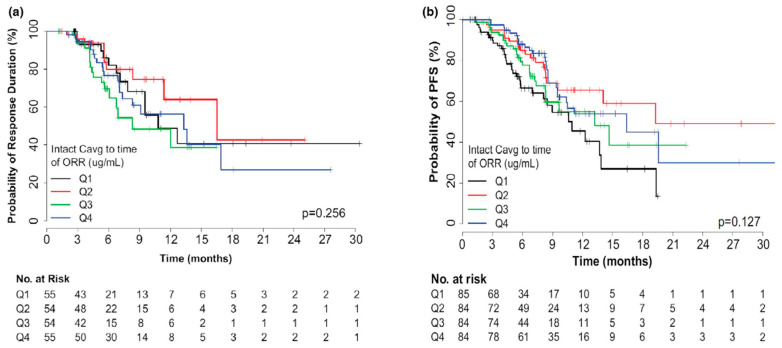
Kaplan–Meier curves showing (**a**) the duration of response and (**b**) progression-free survival stratified by trastuzumab deruxtecan exposure. (Reproduced with permission from Ophelia Yin et al. (2021). Copyright 2021 John Wiley and Sons [122]). Note: C_avg_, average concentration; ORR, objective response rate; PFS, progression-free survival; Q, quartile.

Non-parametric methods assume no particular data distribution, and survival rate results are absolutely based on summary statistics. The Kaplan–Meier (K-M) method is the most popular non-parametric method: the survival time is arranged in ascending order, and the initial sample size, number of occurrences, probability of an occurrence, and survival rate are recorded or calculated for each visit [128]. The survival curve from the K-M method depends completely on observations, though a statistical test, such as the log rank test, can be performed between two survival curves. In an E-R analysis for trastuzumab deruxtecan, a graphical exploration of K-M curves was generated for the time-to-event endpoint (e.g., PFS and duration of response) by quartile (Figure 6) to demonstrate and compare the survival rates of different exposure groups [122]. However, K-M plots are only exploratory and cannot quantify any covariate influence or simulate or predict survival rates for specific scenarios.

As a semi-parametric method, Cox proportional hazards (CPHs) models based on K-M analyses have been extensively applied to investigate the relationship between predictor variables and the survival times of individuals. Unlike non-parametric methods, CPH models can quantify how a covariate affects the hazard rate h(t) at a given time point (Equation (1)) to assess whether ADC exposures significantly affect the hazard ratio [129]. In an E-R analysis for brentuximab vedotin, K-M curves by quartile were developed together with a Cox regression model for PFS [75]. Those results showed that, compared with the positive control, brentuximab vedotin improved PFS in four quantiles of exposure; however, the ADC AUC was not identified as a significant predictor of PFS (*P* = 0.7533) [75].

(1) $$h\;(t)=h_{0}(t)\cdot e^{{(b}_{1}\cdot x_{1}+b_{2}\cdot x_{2}+\cdots +{(b}_{n}\cdot x_{n}))}$$

Although CPH models are widely used, it should be noted that they require key assumptions in model development, for instance that the hazard ratio will remain constant throughout the follow-up period, and each variable provides a linear contribution to the model [129]. Actual covariates and relationships are more complex and time-varying than that [130,131]. To minimize the limitations of CPH models, extended Cox models that incorporate a time-varying hazard rate ratio have been proposed [132]. Yang et al. investigated four commonly used CPH survival models: the proportional intensity model, the Prentice, Williams, and Peterson (PWP) total time model, the PWP gap time model, and a leveraged Poisson regression to analyze ordered recurrent events of the same type. Their results suggest that the two PWP models were robust options for recurrent event analyses [133].

#### 3.2.2. Logistic Regression Models

**Figure 7:**
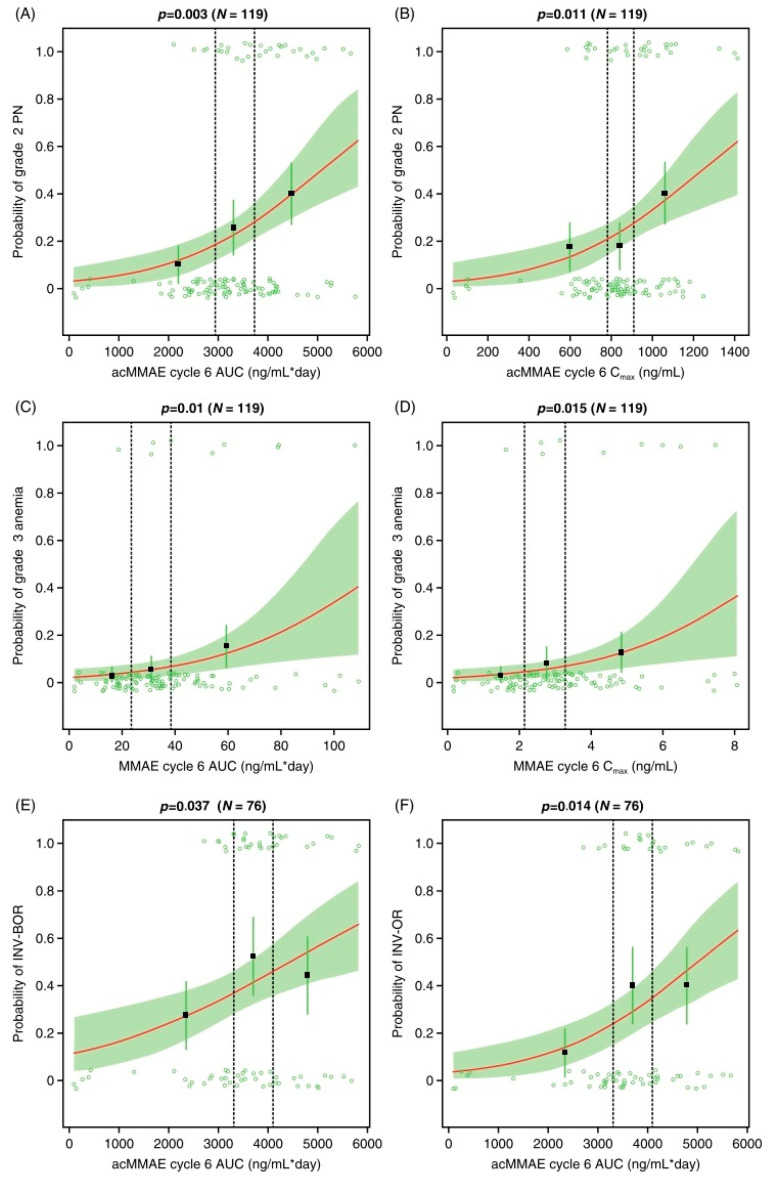
Logistic regression for grade 2 peripheral neuropathy (PN) with the (**A**) AUC and (**B**) C_max_ of acMMAE, grade 3 anemia with the (**C**) AUC and (**D**) C_max_ of released MMAE, and the (**E**) investigator-assessed best overall response (INV-BOR) and (**F**) investigator-assessed overall response (INV-OR) with the AUC of acMMAE. The red solid line represents the logistic regression model prediction; the green shaded area denotes the 90% confidence intervals; the points show the exposure of individual patients with events (*p* = 1) and without events (*p* = 0); black squares and vertical green lines represent the observed fraction of patients with events in each exposure group and 90% confidence intervals for those fractions, respectively; dashed vertical lines signify the bounds of the exposure groups. (Reproduced with permission from Tong Lu et al. (2020). Copyright 2020 Taylor & Francis [137]). Note: acMMAE, antibody-conjugated monomethyl auristatin E; AUC, area under the concentration–time curve; C_max_, maximum concentration; PN, peripheral neuropathy; INV-BOR, investigator-assessed best overall response; INV-OR, investigator-assessed overall response.

Logistic regression (LR) models are popular for analyzing binary outcomes and can be used to predict a binary outcome based on a set of independent variables [134]. LR models were leveraged in E-R analyses for all the approved ADC products to understand the relationship between the exposure level and ADC efficacy and the effects of baseline factors on safety and efficacy responses. For example, in an E-R analysis for gemtuzumab ozogamicin, LR models with a logit link function were developed for the efficacy and safety endpoints of complete remission (CR), CR without platelet recovery (CRp), and veno-occlusive disease (VOD) [135]. Based on the results from that PK-PD model, a clinical utility index that integrated safety and efficacy profiles at a weight ratio of 1 to 1 was calculated using the probability of CR/CRp and VOD. Those results showed that fractionated dosing could provide the best safety and efficacy profile for gemtuzumab ozogamicin and successfully supported re-approval by the U.S. FDA in 2017 and approval by the EMA in 2020 [136]. Exposure–safety and exposure–efficacy relationships for polatuzumab vedotin in relapsed or refractory diffuse large B-cell lymphoma patients were established using LR models. Exposure was modeled by simulating the cycle-6 C_max_ and AUC for antibody-conjugated MMAE (acMMAE) and released MMAE. Supportive studies revealed that both the response rate and safety risk, namely grade 2 peripheral neuropathy or grade 3 anemia, were augmented by exposure, indicating that a dose of not less than 1.8 mg/kg for up to eight cycles provided the best balance of safety and efficacy (Figure 7). The E-R analysis for polatuzumab vedotin furnished a tactic for leveraging E-R consequences from early trials, with an expansive dose scope and longer therapy duration but unidentical drug combinations, and the observed risk–benefit characteristics in the pivotal study with only one dose level to certify the approved label dose. The modeling results for polatuzumab vedotin were accepted by the U.S. FDA and successfully supported its dose regimens [137]. LR model was also applied to investigate E-R relationships in efficacy and safety for mirvetuximab soravtansine, and the results revealed that trough concentration of ADC correlated with efficacy while the AUC of ADC was associated with ocular adverse events. The E-R relationships supported the proposed therapeutic dose regimen [138]. LR model for inotuzumab ozogamicin demonstrated that ADC exposure was prominently correlated with efficacy and safety endpoints [139].

In practice, depending on the purpose of analysis, multiple models are applicable in E-R analysis. For example, in the exposure–safety analysis for polatuzumab vedotin, the probability of binary endpoint best overall response and overall response were modeled using LR models, while time-to-event endpoint was investigated by K-M plots stratified by categories of exposure and CPH models [137]. LR models, K-M plots stratified by exposure quartiles, and CPH models were leveraged to explore the relationship between cycle 1 belantamab mafodotin/MMAF exposure and efficacy or safety endpoints. The integrated benefit–risk assessment demonstrated reduced responses and no reduction in grade ≥3 ocular adverse events/ophthalmic exam findings with an initial dose of 1.9 mg/kg compared to 2.5 mg/kg [140]. The aforementioned models for E-R analysis of belantamab mafodotin also revealed that both efficacy and safety end points were associated with disease factors and patient characteristics, and safety end points were more strongly associated with exposure than efficacy end points, particularly after disease factors and patient characteristics were accounted for in multivariate modeling [141]. E-R analysis for loncastuximab tesirine by K-M, LR, and CPH models were performed to recommend dosing schedule [96]. K-M, LR, and CPH models with cycle 1 exposure metrics and predicted average concentrations from time zero until the end of the cycle in which an event occurred were applied to exposure–safety and exposure–efficacy analyses for tisotumab vedotin, and the relationship between ADC and MMAE exposure and safety end points suggests increased exposure was associated with increased adverse events risk [142]. K-M analysis and CPH model were utilized to establish E-R relationship with model-predicted and observed minimum concentration and area under the concentration–time curve from time zero to day 21 of T-DM1 at cycle 1, revealing that E-R for efficacy was inconsistent across exposure metrics while model-predicted cycle 1 C_min_ showed the strongest E-R trend [117]. A summary of the E-R analysis for ADCs is presented in Table 4.

E-R modeling and interpretation for ADCs face several challenges. One of them is the scarcity of well-conducted dose-ranging trials, which makes the construction of accurate E-R relationships difficult. Datasets from patients treated with a single dose regimen restrict the ability to explore variability in exposures and responses. Sonoko Kawakatsu et al. revealed that multiple dose levels are essential for interpreting the confounding effects of E-R models [143]. ADCs exhibit time-dependent clearance with increased drug exposure over time at a constant dose due to time-varying disease status, which complicates the quantification and interpretation of the E-R relationship [144]. Baseline prognostic factors, such as disease severity (e.g., Eastern Cooperative Oncology Group status), can confound E-R analyses. Sicker patients might carry a greater tumor burden that increases drug clearance, resulting in lower exposure levels. Such factors can elicit artificial E-R relationships, overestimating the benefit of higher drug exposure [116,122]. Prognostic disease factors and baseline disease burden could particularly affect the interpretation of the E-R relationship, so they should always be taken into consideration in E-R analyses [116]. However, traditional E-R methods are inadequate to account for the dynamicity of drug exposure and disease progression, and different exposure metrics have their limitations, which are discussed in Section 3.1. Immunogenicity to ADCs can potentially affect their PK, PD, safety, and efficacy in humans; therefore, monitoring immunogenicity is a pivotal component in drug development [145]. Immunogenicity is usually neglected in exploring the E-R relationship for ADC except for brentuximab vedotin, and the results revealed that positive antidrug antibody status significantly augmented brentuximab vedotin clearance [75]. It is suggested that the influence of immunogenicity on clinical response should be paid enough concern in E-R analyses. These challenges highlight the urgent need for methodological advances and robust trial designs to accurately quantify E-R relationships.

**Table 4:** E-R analyses for ADCs.

Agent	Components	Indication	Approval Year and Regulatory Agencies	Model Type	Key Endpoints	Tested and Revealed * Exposure Metrics	Software	Purpose	Reference
Gemtuzumab ozogamicin	Anti-CD33 mAb, calicheamicin, hydrazone disulfide linker	CD33-positive acute myeloid leukemia	2000, US FDA and EMA	LR model	Efficacy endpoints: CR/CRp. Safety endpoints: VOD, grade ≥ 3 hepatic adverse events.	Tested: model predicted cycle 1 C_max_, overall C_max_, and overall AUC for total antibody. Revealed: cycle 1 C_max_ for total antibody.	R 3.2.2, NONMEM 7.3 and Perl-speaks-NONMEM 4.2.0	To provide a rationale for new fractionated dosing regimens.	[135]
Brentuximab vedotin	Anti-CD30 mAb, MMAE, vc linker	Hodgkin lymphoma, anaplastic large cell lymphoma	2011, US FDA	K-M plot, CPH and LR model	Efficacy endpoints: ORR. Safety endpoints: grade ≥ 2 peripheral neuropathy, grade ≥ 3 neutropenia and thrombocytopenia.	Tested: model predicted C_trough,ss_ and AUC_ss_ for ADC and payload. Revealed: AUC_ss_ for ADC.	R 3.2.0	To support the starting dose and dose reduction.	[75]
Trastuzumab emtansine	Trastuzumab, DM-1, thioether linker	HER2-positive, metastatic breast cancer	2013, US FDA	K-M plot, CPH and LR model	Efficacy endpoint: OS, PFS, ORR. Safety endpoints: grade ≥ 3 adverse event/thrombocytopenia/hepatotoxicity.	Tested: model predicted and observed C_min_ and AUC at cycle 1 for ADC. Revealed: cycle 1 C_min_ for ADC.	Splus 8.2	To support the dose regimen.	[117]
Inotuzumab Ozogamicin	Inotuzumab, *N*-acetyl-calicheamicin 1, 2-dimethyl hydrazine dichloride, acid-cleavable linker	Relapsed or refractory B-cell precursor acute lymphoblastic leukemia	2017, US FDA	LR model	Efficacy endpoints: CR/CRi, MRD-negativity. Safety endpoints: hepatic event, VOD/SOS, and grade ≥ 3 AEs.	Tested: model predicted C_max,event_, C_max,overall_, cAUC, C_avg_, and cAUCP1 for ADC. Revealed: ADC C_avg_ and cAUCP1 for ADC.	R 3.0.2	To support dose modification in clinic.	[139]
Polatuzumab vedotin	Anti-CD79b mAb, MMAE, vc linker	Relapsed or refractory diffuse large B-cell lymphoma	2019, US FDA	K-M plot, CPH and LR model	Efficacy endpoints: DOR, PFS, OS, OR. Safety endpoints: grade ≥ 3 hepatotoxicity/neutropenia/infections and infestations/anemia/thrombocytopenia/peripheral neuropathy, dose modification due to AE.	Tested: model predicted cycle 6 AUC and C_max_ for ADC and payload. Revealed: cycle 6 AUC for ADC.	Not reported	To assess the risk/benefit.	[137]
Enfortumab vedotin	Anti–Nectin-4 mAb, MMAE, vc linker	Locally advanced/metastatic urothelial carcinoma	2019, US FDA	K-M plot, CPH and LR model	Efficacy endpoints: OS, BOR. Safety endpoints: grade ≥ 3 TEAE/rash/severe cutaneous AE/hyperglycemia, dose adjustment, grade ≥ 2 peripheral neuropathy.	Tested and revealed: model predicted C_avg,last_ for ADC and payload.	SAS 9.4 and R 3.6.2	To support the starting dose regimen.	[98]
Trastuzumab deruxtecan	Anti-HER2 mA, DXd, tetrapeptid linker	HER2-positive breast cancer, gastric cancer, and non-small cell lung cancer	2019, US FDA	K-M plot, CPH and LR model	Efficacy endpoints: ORR, PFS, DOR. Safety endpoints: ILD.	Tested: model predicted C_max_, C_min_, AUC at cycle 1 and steady-state, C_avg_ during treatment and C_avg,last_ for ADC and payload. Revealed: C_avg,last_ for ADC.	Not reported	To characterize E-R relationship and evaluate effect of covariates.	[122]
Belantamab mafodotin	Anti-B-cell maturation antigen mAb, MMAF, maleimidocaproyl linker	Elapsed or refractory multiple myeloma	2020, US FDA	K-M plot, CPH and LR model	Efficacy endpoints: PFS, PoR, TTR, TTBR, and DOR. Safety endpoints: probability of grade ≥ 2 or ≥3 corneal events and time to first event of grade ≥ 2 or ≥3 corneal events.	Tested: model predicted cycle 1 C_max_, C_avg_, and C_tau_ for ADC, cycle 1 C_max_ and C_avg_ for cys-mcMMAF. Revealed: cycle 1 C_avg_ for ADC.	R	To support dose selection	[140,141]
Sacituzumab Govitecan	Antibody against Trop-2, SN-38, hydrolyzable linker	Triple-negative breast cancer, urothelial cancer	2022, US FDA	LR	Efficacy endpoints: PSF, OS. Safety endpoints: nausea/vomiting and diarrhea of any grade.	Tested: model predicted cycle 1 AUC and C_max_ for ADC, total antibody and payload. Revealed: cycle 1 AUC and C_max_ for payload.	Postgre SQL 14.4 and R 4.2.0	To explore the risk factors associated with adverse events.	[146,147]
Loncastuximab tesirine	Anti-CD19 antibody, SG3199, valine-alanine linker	Diffuse large B-cell lymphoma	2021, US FDA	K-M, CPH and LR model	Efficacy endpoints: OS, PFS, and DoR. Safety endpoints: grade ≥ 2 TEAE.	Tested: model predicted C_max_, C_min_, and AUC at first 3 cycles for ADC, total antibody, and payload. Revealed: cycle 1 C_avg_ for ADC.	R 4.0.1 and SAS 9.4	To support dose regimen.	[96]
Tisotumab vedotin	Tissue factor specific mAb, MMAE, vc linker	Recurrent or metastatic cervical cancer	2021, US FDA	K-M plot, CPH and LR model	Efficacy endpoints: ORR, PFS, OS, DOR. Safety endpoints: grade ≥ 2 ocular AEs/peripheral neuropathy/bleeding AEs, grade ≥ 3 AEs, treatment-related dose modifications, all SAEs, and treatment-related SAEs.	Tested: model predicted cycle 1 AUC, C_max_, and C_trough_ for MMAE; cycle 1 AUC and C_max_ for ADC; C_avg,last_ for MMAE and ADC. Revealed: cycle 1 AUC, C_max_, and C_avg,last_ for ADC; cycle 1 AUC for MMAE.	R 4.0.2	To support the dose schedule.	[142]
Mirvetuximab soravtansine	Anti-folate receptor α mAb, DM4, sulfo-SPDB linker	Folate receptor α positive, platinum-resistant ovarian cancer	2022, US FDA	K-M plot, CPH and LR model	Efficacy endpoints: ORR, PFS, OS. Safety endpoints: ocular AEs (as well as the time to onset of ocular AEs) and peripheral neuropathy.	Tested: model predicted C_max_, C_trough_, and AUC at cycle 1 for ADC, DM4, and S-methyl-DM4. Revealed: C_trough_ and AUC at cycle 1 for ADC.	Not reported	Justification of therapeutic dose regimen.	[138]

Note: AE: adverse event; AUC: area under the concentration–time curve; AUC_ss_: area under the concentration–time curve at steady state; cAUC: cumulative area under the concentration–time curve; cAUCP1: cumulative area under the concentration–time curve in the first cycle of treatment; C_avg_: average concentration calculated as the ratio of cAUC to its corresponding timeframe; C_avg,last_: average concentrations from time zero until end of the cycle in which an event occurred; C_max,event_: maximum observed concentration prior to response; C_max,overall_: maximum observed concentration for the duration of treatment; C_tau_: concentration at the end of the dosing interval; C_trough,ss_: trough concentration at steady state; CPH: Cox proportional hazard; CR/CRi: complete remission/complete remission with incomplete hematologic recovery; CR/CRp: complete remission/complete remission without platelet recovery; DoR: duration of response; ILD: interstitial lung disease; MRD, minimal residual disease; OR: objective response; ORR: objective response rate; OS: overall survival; PFS: progression-free survival; PoR: probability of response; SAE: serious adverse event; TEAE: treatment-emergent adverse events; TTBR: time to best response; TTR: time to response; VOD/SOS: Veno-occlusive disease/sinusoidal obstruction syndrome. * revealed exposure metrics here refer to those demonstrating the strongest exposure–response trends.

## 4. Conclusions and Perspectives

ADCs have emerged as a solid therapeutic tactic for different types of malignancies. By integrating an mAb and cytotoxic agent, ADCs have revolutionized cancer therapy by precisely targeting carcinoma cells, which prominently enhances therapeutic efficacy and reduces off-target toxicity. Despite the great progress already made, both the development and clinical application of ADCs face great challenges, including an in-depth and comprehensive understanding of their PK properties. The complex PK of ADCs results from their intricate distribution, binding, and elimination, as well as the deconjugation process and resulting need for PK profiles for the released payload. The PK profiles for ADCs need to reflect the mAb framework, characteristics of the payload, linker stability, DAR, antigen expression, tumor volume, cancer heterogeneity, and so on. All those factors collectively dictate the distribution, catabolism, and elimination processes for ADCs in vivo and ultimately influence their efficacy and safety.

The development of proper models for ADCs is essential for predicting whole-body PK, especially in tumors; describing the affinity of an ADC for its target, the kinetics of deconjugation, intracellular processing, and efflux from cancer cells; assessing the sources responsible for inter-individual variability in PK; and predicting DDI and drug exposure to optimize dose regimens in the clinic. In this review, we have presented an overview of modeling strategies for PBPK models, semi-mechanistic and mechanistic PK models, population PK models, and E-R analyses for ADCs and discussed their specific applications in preclinical and clinical trials. Although these models can deeply describe the complicated PK profiles of ADCs, some of them, e.g., PBPK models, encounter the challenges of accurately calculating intricate parameters, high inter-individual variability, and a degree of fitting bias. Moreover, accessible data are the foundation of PK modeling. However, ADCs quantification in tumors poses a huge challenge, and the limitations of sampling and analytical technology restrict the acquisition of intact ADCs, deconjugated ADCs, ADCs with different DARs, and cytotoxic agent concentrations in tumors. The inaccessibility of tumor concentrations undermines the ability of the models to describe the complicated processes occurring in tumors. Currently, ligand-binding assays (LBAs) and hybrid LC-MS/MS are the techniques most widely applied in ADC analyses. LBAs with low specificity and sensitivity are only leveraged to measure total antibodies; they are incompetent in quantifying ADCs with different DARs [148]. LBA-LC–MS/MS and immunocapture or bead-based affinity capture LC-MS/MS have been developed to quantify DAR values for conjugated antibodies [149]. However, real-time DAR monitoring remains arduous because of the time-consuming specimen pretreatment required. Advances in analytical technology for ADCs might enable the development of reliable models based on fruitful concentration data for ADCs and their catabolites and metabolites. Collectively, full elucidation of in vivo behavior for ADCs based on development of basic research on tumor biology and cellular disposition of ADCs is essential for the establishment of mechanism-based model. Artificial intelligence and machine learning can be leveraged in construction of ADCs models.

Despite advances in PK-PD investigations for ADCs, several limitations remain unresolved. First, clinical research has concentrated on E-R analyses based on population PK models, with a relative scarcity of mechanism-based PK-PD models for ADCs. Although some semi-mechanistic PK models incorporate the kinetics needed for DAR value degradation, they are predominantly empirical rather than mechanistic. In contrast, mechanism-based PK-PD models are needed to comprehensively describe ADC binding to its antigen, internalization, deconjugation, and catabolism; the distribution of ADCs and their cytotoxic agents within and beyond the tumor; the metabolism and elimination of the payload; the synergistic antitumor efficacy of ADCs and their payloads; and the potential for DDIs. Second, the catabolism and PD of antibodies are not usually fully elucidated in proposed models. Third, individualized therapy should take antigen expression, tumor volume, and cancer heterogeneity into account based on oncology biology, pathology, and clinical stage. Fourth, the interpretation and complexity of the developed models needs to be seriously considered. Lastly, well-designed and conducted clinical trials are essential for reliable concentration and response data, which is the cornerstone for modeling, while sparse and even missing data may result in a greater magnitude of variability in parameters.

In conclusion, the relatively complex structure and acting mechanisms of ADCs pose huge challenges to fully elucidate their PK and PD. This review has presented an overview of diverse modeling approaches used for ADCs, highlighting the essential role of PK and PD models in elucidating the PK profiles and acting mechanisms and optimizing dosing regimens. Deeper investigations of the mechanistic actions of ADCs will enable the construction of more mechanism-based PK-PD models to support the research and development of innovative ADCs and optimize drug regimens.

## Figures and Tables

**Table 1:** PBPK models for ADCs.

Agent	Components	DAR	Indication	Approval Year and Regulatory Agencies	Software	Purpose	Reference
Pinatuzumab vedotin	anti-CD22 mAb, MMAE, vc linker	3.5	None	Not approved	Simcyp version 12	Prediction of MMAE-based DDI potential	[33]
Enfortumab vedotin	anti-Nectin-4 mAb, MMAE, vc linker	3.7	Locally advanced/metastatic urothelial carcinoma	2019, US FDA	Simcyp version 19	Prediction of MMAE-based DDI potential	[34]
Polatuzumab Vedotin	Anti-CD79b mAb, MMAE, vc linker	3.7	relapsed or refractory diffuse large B-cell lymphoma	2019, US FDA	Simcyp version 12	Prediction of DDI potential	[35]
Trastuzumab-vc-MMAE	Trastuzumab, MMAE, vc linker	4.5	None	Not approved	Berkeley Madonna	Quantification of whole-body disposition of MMAE	[38]
Trastuzumab emtansine	Trastuzumab, DM-1, thioether linker	3.5	HER2-positive, metastatic breast cancer	2013, US FDA	ADAPT V, Berkeley Madonna, IQMTools	Prediction of whole-body disposition of ADCs, and translation into human.	[41]
Trastuzumab emtansine	Trastuzumab, DM-1, thioether linker	3.5	HER2-positive, metastatic breast cancer	2013, US FDA	MATLAB	Modeling both the systemic and tissue-scale distributions of antibodies and ADCs.	[45]

**Table 2:** Semi-mechanistic and mechanistic-based PK models for ADCs.

Agent	Components	DAR	Indication	Approval Year and Regulatory Agencies	Model Type	Software	Purpose	Reference
Trastuzumab Emtansine	Trastuzumab, DM-1, thioether linker	3.5	HER2-positive, metastatic breast cancer	2013, US FDA	Mechanistic PK	NONMEM 7.2	To Elucidate ADC PK and quantify rates of payload deconjugation.	[39]
Anti-HER2-vc-MMAF, anti-NaPi2b-vc-MMAE, anti-STEAP1-vc-MMAE	Anti-HER2 mAb, Anti-NaPi2b mAb, Anti-STEAP1 mAb, MMAF, MMAE, vc linker	Not obtained for Anti-HER2-vc-MMAF, 3-4 for anti-NaPi2b-vc-MMAE and anti-STEAP1-vc-MMAE	None	Not approved	Mechanistic PK	SimBiology^®^	To describe and predict PK of ADCs with VC linker linked to MMAF or MMAE.	[54]
Trastuzumab emtansine	Trastuzumab, DM-1, thioether linker	3.5	HER2-positive, metastatic breast cancer	2013, US FDA	Mechanistic PK	Not reported	To characterize ADC PK based on TMDD model.	[55]
Anti-CD79b-MMAE	Anti-CD79 mAb, MMAE, vc linker	3.5	Diffuse large B cell lymphoma	2019, US FDA	Semi-mechanistic PK	S-ADAPT II 1.57	To simultaneously describe PK of multiple analytes.	[56]
Brentuximab vedotin	Anti-CD30 mAb, MMAE, vc linker	4.4	Hodgkin lymphoma, anaplastic large cell lymphoma	2011, US FDA	Mechanistic PK	ADAPT-5, Berkeley Madonna	Preclinical to clinical translation of ADC efficacy.	[52]
A1mcMMAF	anti-5T4 antibody (A1), MMAF, maleimidocaproyl linker.	4	None	Not approved	Mechanistic PK	ADAPT-5, Berkeley Madonna	To better understand the underlying processes responsible for the disposition of ADCs.	[58]
Trastuzumab emtansine	Trastuzumab, DM-1, thioether linker	3.5	HER2-positive, metastatic breast cancer	2013, US FDA	Mechanistic PK	Berkeley Madonna, ADAPT-5	To predict preclinical pumor PK	[59]
Trastuzumab deruxtecan	Anti-HER2 mA, DXd, tetrapeptid linker	8	HER2-positive breast cancer, gastric cancer and non-small cell lung cancer	2019, US FDA	Mechanistic PK	Phoenix 8.3.3, NONMEM 7.4	To understand the relationship between antigen expression and downstream efficacy outcomes.	[61]
Trastuzumab-vc-MMAE	Trastuzumab, MMAE, vc linker	4	None	Not approved	Semi-mechanistic PK	ADAPT 5, MATLAB	To evaluate the effect of antigen expression level on ADC exposure in solid tumor.	[62]
Inotuzumab Ozogamicin	Inotuzumab, *N*-acetyl-calicheamicin 1, 2-dimethyl hydrazine dichloride, acid-cleavable linker	3.5	Relapsed or refractory B-cell precursor acute lymphoblastic leukemia	2017, US FDA	Mechanistic PK	JACOBIAN Modeling and Optimization Software	Preclinical to clinical translation.	[63]
Trastuzumab emtansine	Trastuzumab, DM-1, thioether linker	3.5	HER2-positive, metastatic breast cancer	2013, US FDA	Mechanistic PK	Berkeley Madonna, ADAPT-5	Clinical translation.	[64]
Anti-glypican 3 ADC	Anti-glypican 3 mAb, tubulysin, vc linker	3	None	Not approved	Semi-mechanistic PK	Pheonix 6.4 NLME 1.3	Translation of preclinical efficacy of ADC to human.	[65]
PF-06804103	Anti-HER2 mAb, auristatin 101, vc linker	4	None	Not approved	Mechanism-based PK	Not reported	Quantitative comparison of PF- 06804103 and T-DM1 in terms of their PK and efficacy.	[67]
T-DM1, T-SPP-DM1	Trastuzumab, DM-1, thioether or disulfide linker	4.0 for T-DM1, 3.2 for T-SPP-DM1	HER2-positive, metastatic breast cancer	2013, US FDA	Mechanistic PK-PD	R 2.10.0	To explore the mechanistic processes of ADC PK, tumor uptake, catabolism, and tumor response.	[68]

**Table 3:** Population PK models for ADCs.

Agent	Components	DAR	Indication	Approval Year and Regulatory Agencies	Model Type	Software	Purpose	Reference
Trastuzumab Emtansine	Trastuzumab, DM-1, thioether linker	3.5	HER2-positive, metastatic breast cancer	2013, US FDA	Single-analyte-based population PK	NONMEM	To understand clinical factors that might affect exposure.	[70,71,72]
Inotuzumab Ozogamicin	Inotuzumab, *N*-acetyl-calicheamicin 1, 2-dimethyl hydrazine dichloride, acid-cleavable linker	3.5	Relapsed or refractory B-cell precursor acute lymphoblastic leukemia	2017, US FDA	Single-analyte-based population PK	NONMEM	Dose adjustment.	[73,74]
Brentuximab vedotin	Anti-CD30 mAb, MMAE, vc linker	4.4	Hodgkin lymphoma, anaplastic large cell lymphoma	2011, US FDA	Two-analyte-based population PK	NONMEM	Identification of covariates influencing PK and PK variability.	[75,76,78,79,80]
Trastuzumab deruxtecan	Anti-HER2 mAb, DXd, tetrapeptid linker	8	HER2-positive breast cancer, gastric cancer, and non-small cell lung cancer	2019, US FDA	Two-analyte-based population PK	NONMEM	Identification of covariates influencing PK.	[81]
Trastuzumab emtansine	Trastuzumab, DM-1, thioether linker	3.5	HER2-positive, metastatic breast cancer	2013, US FDA	Two-analyte-based population PK	NONMEM	To understand PK profiles and identify influence of patient characteristics on PK.	[82,83]
Camidanlumab tesirine	Anti-CD25 mAb, SG3199, cleavable linker	2.3	None	Not approved	Two-analyte-based population PK	NONMEM	To characterize PK profile and identify covariates influencing PK	[84]
Polatuzumab Vedotin	Anti-CD79b mAb, MMAE, vc linker	3.7	Relapsed or refractory diffuse large B-cell lymphoma	2019, US FDA	Two-analyte-based population PK	NONMEM	Description of antibody-conjugated and free MMAE concentration, and examination dosing decisions.	[85,86]
Tisotumab vedotin	Tissue factor specific mAb, MMAE, vc linker	4	Recurrent or metastatic cervical cancer	2021, US FDA	Two-analyte-based population PK	NONMEM	To assess the PK profile of tisotumab vedotin and MMAE.	[89]
FS-1502	Anti-HER2 antibody, MMAF, a β-glucuronidase cleavable linker	2	None	Not approved	Two-analyte-based population PK	NONMEM	Dose selection	[90]
Belantamab mafodotin	Anti-B-cell maturation antigen mAb, MMAF, maleimidocaproyl linker	4	Elapsed or refractory multiple myeloma	2020, US FDA (withdrawn)	Three-analyte-based population PK	NONMEM	To identify disease factors and patient characteristics impacting PK parameters and exposure.	[92,93,94]
Loncastuximab tesirine	Anti-CD19 antibody, SG3199, valine-alanine linker	2.3	Diffuse large B-cell lymphoma	2021, US FDA	Three-analyte-based population PK	NONMEM	To assess the influence of covariates on PK, and to explore E-R relationships.	[96]
Depatuxizumab mafodotin	Recombinant IgG1κ antibody, MMAF, maleimido-caproyl linker	3 and 4 for different manufacture	None	Not approved	Three-analyte-based population PK	NONMEM	Simultaneous description of concentration–time for ADC, total antibody, and payload.	[97]
Enfortumab vedotin	Anti–Nectin-4 mAb, MMAE, vc linker	3.7	Locally advanced/metastatic urothelial carcinoma	2019, US FDA	Three-analyte-based population PK	NONMEM	To assess the influence of covariates on PK and exposure, and E-R analysis.	[98]
Gemtuzumab ozogamicin	Anti-CD33 mAb, calicheamicin, hydrazone disulfide linker	2-3	CD33-positive acute myeloid leukemia	2000, US FDA	Three-analyte-based population PK	NONMEM	To explore intrinsic and extrinsic factors that may influence exposure.	[99,100]
Mirvetuximab soravtansine	Anti-folate receptor α mAb, DM4, sulfo-SPDB linker	3.4	Folate receptor α positive, platinum-resistant ovarian cancer	2022, US FDA	Three-analyte-based population PK	NONMEM	To describe PK profiles, and assess the influence of covariates on PK and exposure.	[102]
Patritumab deruxtecan	mAb against HER-3, deruxtecan, and a tetrapeptide-based linker	Not obtained	None	Not approved	Three or two-analyte-based population PK	NONMEM	To assess the influence of covariates on PK and exposure.	[104,105]
Sacituzumab govitecan	Antibody against Trop-2, SN-38, hydrolyzable linker.	8	Triple-negative breast cancer, urothelial cancer	2022, US FDA	Three-analyte-based population PK	NONMEM	To assess the influence of covariates on PK and exposure.	[107]

## Data Availability

No new data were created or analyzed in this study.

## Abbreviations

acMMAE	antibody-conjugated monomethyl auristatin E
ADCs	antibody–drug conjugates
ADME	absorption, distribution, metabolism, and excretion
ALL	acute lymphocytic leukemia
AUC	area under the concentration–time curve
CDE	China Center for Drug Evaluation
C_avg,C1_	average concentration in the first cycle
C_avg,ss_	average steady state concentration
C_avg,TE_	average concentration up to an event time
C_max_	maximum concentration
CPH	Cox proportional hazards
CR	complete remission
CRp	CR without platelet recovery
C_trough,C1_	trough concentration in the first cycle
C_trough,ss_	steady state trough concentration
CYP	cytochrome P450
DAR	drug-to-antibody ratio
EGFR	epithelial growth factor
E-R	exposure-response
HER-2	human epidermal growth factor receptor 2
K-M	Kaplan–Meier
LBAs	ligand-binding assays
LC-MS	liquid chromatography–mass spectrophotometry
LR	logistic regression
mAbs	monoclonal antibodies
MMAE	monomethyl auristatin E
MMAF	monomethyl auristatin F
NHL	non-Hodgkin’s lymphoma
NMPA	National Medicine Products Agency
PD	pharmacodynamics
PFS	progression-free survival
PK	pharmacokinetic
PK/PD	pharmacokinetics/pharmacodynamics
PWP	Prentice, Williams and Peterson
T-DM1	trastuzumab emtansine
TMDD	target-mediated drug disposition
vc	valine-citrulline
VOD	veno-occlusive disease
